# Deciphering the Transcriptomic Signatures of Aging Across Organs in Mice

**DOI:** 10.1111/acel.70357

**Published:** 2026-01-08

**Authors:** Sarah Morsy, Enzo Scifo, Kan Xie, Kristina Schaaf, Jenny Russ, Stefan Paulusch, Elena De Domenico, Paolo Salomoni, Daniele Bano, Dan Ehninger

**Affiliations:** ^1^ Translational Biogerontology Lab, German Center for Neurodegenerative Diseases (DZNE) Bonn Germany; ^2^ Nuclear Function Lab, German Center for Neurodegenerative Diseases (DZNE) Bonn Germany; ^3^ Systems Medicine, German Center for Neurodegenerative Diseases (DZNE) Bonn Germany; ^4^ PRECISE Platform for Single Cell Genomics and Epigenomics (PRECISE), German Center for Neurodegenerative Diseases (DZNE), University of Bonn, Bonn Germany; ^5^ Genomics and Immunoregulation, Life & Medical Sciences Institute (LIMES), University of Bonn Bonn Germany; ^6^ Aging and Neurodegeneration Lab, German Center for Neurodegenerative Diseases (DZNE) Bonn Germany

**Keywords:** aging, mouse organs, qPCR, trajectories, transcriptomics

## Abstract

Aging, a major risk factor for numerous diseases, is associated with significant transcriptional changes across organs. However, the age of onset, extent of transcriptomic changes and how they unfold are not fully understood. We performed bulk RNA sequencing on eight organs (brain, heart, kidney, liver, lung, skeletal muscle, spleen, and testis) from male C57BL/6J mice across much of the murine lifespan covering 3‐, 5‐, 8‐, 14‐, 20‐ and 26‐month‐old animals. Our analysis revealed that age‐related transcriptomic shifts vary in both timing and extent, with early shifts in lung, spleen, and testis; mid‐life changes in heart, kidney, and skeletal muscle; and later alterations in brain and liver. The extent of age‐related transcriptomic changes ranged from very low (testis) to high (kidney, liver, spleen). A linear mixed‐effects model identified genes with tissue‐specific aging trajectories. By integrating hub gene analysis and functional enrichment, we uncovered aging signatures that are either tissue‐specific or shared across multiple organs, including those related to immune response, mitochondrial dysfunction, extracellular matrix remodeling, and cellular senescence. This study provides a systems‐level resource for advancing aging research.

## Graphical abstract

Comprehensive transcriptomic profiling of eight mouse organs across six ages reveals how aging reshapes biological processes. Trajectory and network analyses distinguish shared and organ‐specific signatures, charting the molecular landscape of systemic aging.

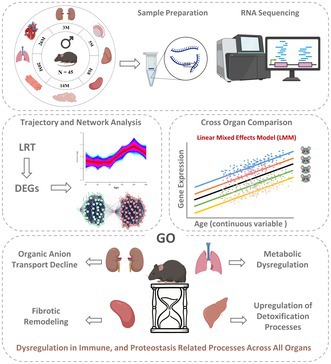

## 1 Introduction

Aging is characterized by a progressive accumulation of molecular damage across multiple biological levels‐cells, tissues, and organs‐culminating in functional decline, increased disease susceptibility, and ultimately death (Gladyshev 2013). Although various hypotheses have been proposed to explain the biological underpinnings of aging, the precise molecular mechanisms driving these changes remain incompletely understood (Keshavarz, Xie, Bano, and Ehninger 2023; Keshavarz, Xie, Schaaf, et al. 2023; Lopez‐Otin et al. 2013). Specifically, the key drivers and heterogeneity of transcriptomic changes across organs, their extent or age of onset, have not been sufficiently explored.

In recent years, high‐throughput omics technologies‐including genomics, epigenomics, transcriptomics, and proteomics‐have been increasingly leveraged to investigate the complex, multifactorial nature of aging (Davie et al. 2018; Schaum et al. 2020; Sen et al. 2016; Takasugi et al. 2024; Zou et al. 2000). Among these, transcriptomic profiling has been particularly informative in uncovering age‐related gene expression changes. However, many rodent transcriptomic studies have been restricted to single‐organ analyses (Graber et al. 2023; Takemon et al. 2021; White et al. 2015), thereby limiting their ability to capture systemic aging signatures or inter‐organ aging dynamics. A transcriptomic study of aging kidney revealed that immune‐related genes were upregulated, while those encoding heat shock proteins were downregulated (Takemon et al. 2021). Similarly, genes associated with immune response and lipid metabolism were upregulated and downregulated, respectively, in the mouse liver (White et al. 2015). Moreover, a recent report determined downregulation of genes related to redox balance, mitochondrial electron transport chain (ETC), and upregulation of genes encoding heat shock proteins in the aging mouse skeletal muscle (Staunton et al. 2022).

Despite these tissue‐specific findings, comprehensive multi‐organ insights into systemic aging remain limited. Notably, a large‐scale study by Schaum et al. identified inflammatory, mitochondrial, and proteostasis‐related gene expression changes shared across several organs. In addition, they reported variability in the onset and intensity of transcriptomic changes between organs (Schaum et al. 2020). Similarly, Shavlakadze et al. demonstrated inter‐organ enrichment of inflammatory and mitochondrial pathways in aging rats (Shavlakadze et al. 2019). Even when multi‐tissue studies were conducted, they often suffered from small sample sizes or limited age coverage, reducing statistical power and the ability to resolve complex expression trajectories over time (Schaum et al. 2020; Takasugi et al. 2024).

Additionally, the common reliance on pairwise comparisons between specific age groups in many studies imposes methodological constraints. While pairwise comparisons can identify differences between selected time points, they are limited in their ability to detect gradual, non‐linear, or late‐onset transcriptional changes over time. This approach can also introduce biases based on the arbitrary selection of comparison groups, potentially obscuring broader aging patterns.

Here, we performed bulk RNA sequencing of eight major organs‐brain, heart, kidney, liver, lung, skeletal muscle, spleen, and testis‐harvested from male C57BL/6J mice at six age stages spanning young adulthood to late life (3, 5, 8, 14, 20, and 26 months). We selected these organs based on their known vulnerability to aging in both mice and humans, spanning multiple physiological systems.

Toward addressing some of the shortcomings from previous studies and capturing overarching age‐associated transcriptomic dynamics, we employed an experimental design with a large sample size (*N* = 45) and profiled changes using the likelihood ratio test (LRT) framework. Unlike pairwise comparisons which assess significance of changes at any two levels of a given factor, the LRT provides an overall measure of significance for differences across all levels and therefore minimizes potential biases arising from any two levels (Love et al. 2014).

By integrating clustering, trajectory, and network analyses, we further dissected common as well as organ‐dominant transcriptional changes and key gene hubs associated with aging. This system‐wide approach provides a comprehensive view of transcriptomic aging signatures and offers novel insights into molecular features associated with aging across tissues in mice. Interestingly, the onset of age‐associated transcriptomic changes varied considerably across organs. Early transcriptional shifts were observed in the lung, spleen, and testis, whereas the brain and liver demonstrated a delayed onset. The heart, kidney, and skeletal muscle displayed intermediate, mid‐life activation of aging‐related transcriptional programs. Our analysis revealed that the liver, kidney, and spleen exhibited the most pronounced age‐associated transcriptomic changes compared to the other organs. Transcriptomic changes in the other organs were either modest (lung, heart, skeletal muscle) or minimal (brain and testis). In both the liver and spleen, transcriptomic profiles remained relatively stable from young adulthood to mid‐life. In contrast, the kidney showed a more variable pattern, with fluctuations in gene expression from young adulthood to mid‐life, followed by a rapid shift in late life and a subsequent decline at the oldest age.

To better capture the complexity of aging across tissues, we employed linear mixed‐effects modeling (LMM) to identify genes whose age‐related expression trajectories significantly differ between organs. Unlike traditional approaches that assess each tissue in isolation, this integrative model jointly analyzes data across tissues while accounting for matched sampling from individual animals. By incorporating a random intercept for each mouse and testing the interaction between age and tissue, LMM enables the detection of tissue‐specific transcriptomic aging dynamics. This approach offers greater statistical rigor and power in identifying divergent and convergent aging signatures across organs, highlighting organ‐specific regulatory programs and shared systemic responses.

Consistent with proposed hallmarks of aging, signatures of immune activation/inflammatory responses, extracellular matrix (ECM) remodeling and mitochondrial dysfunction emerged as shared transcriptomic features across most organs, except for the testis. Additionally, cellular senescence‐related pathways were particularly enriched in the kidney, liver, and spleen. Beyond these shared patterns, we identified distinct organ‐enriched aging signatures. For instance, the liver showed dysregulated lipid metabolism; the lung was characterized by changes in detoxification pathways, while the spleen exhibited transcriptional alterations related to ribosome biogenesis and loss of proteostasis.

Complementing these findings, network analysis of differentially expressed genes (DEGs) identified enriched gene hubs within each organ. These hubs not only corroborated the trajectory‐based insights but also highlighted potential key drivers of aging at the organ level, offering new targets for mechanistic exploration.

## 2 Results

### 2.1 Diverse Age‐Associated Differential Gene Expression Patterns Across Organs

**FIGURE 1:**
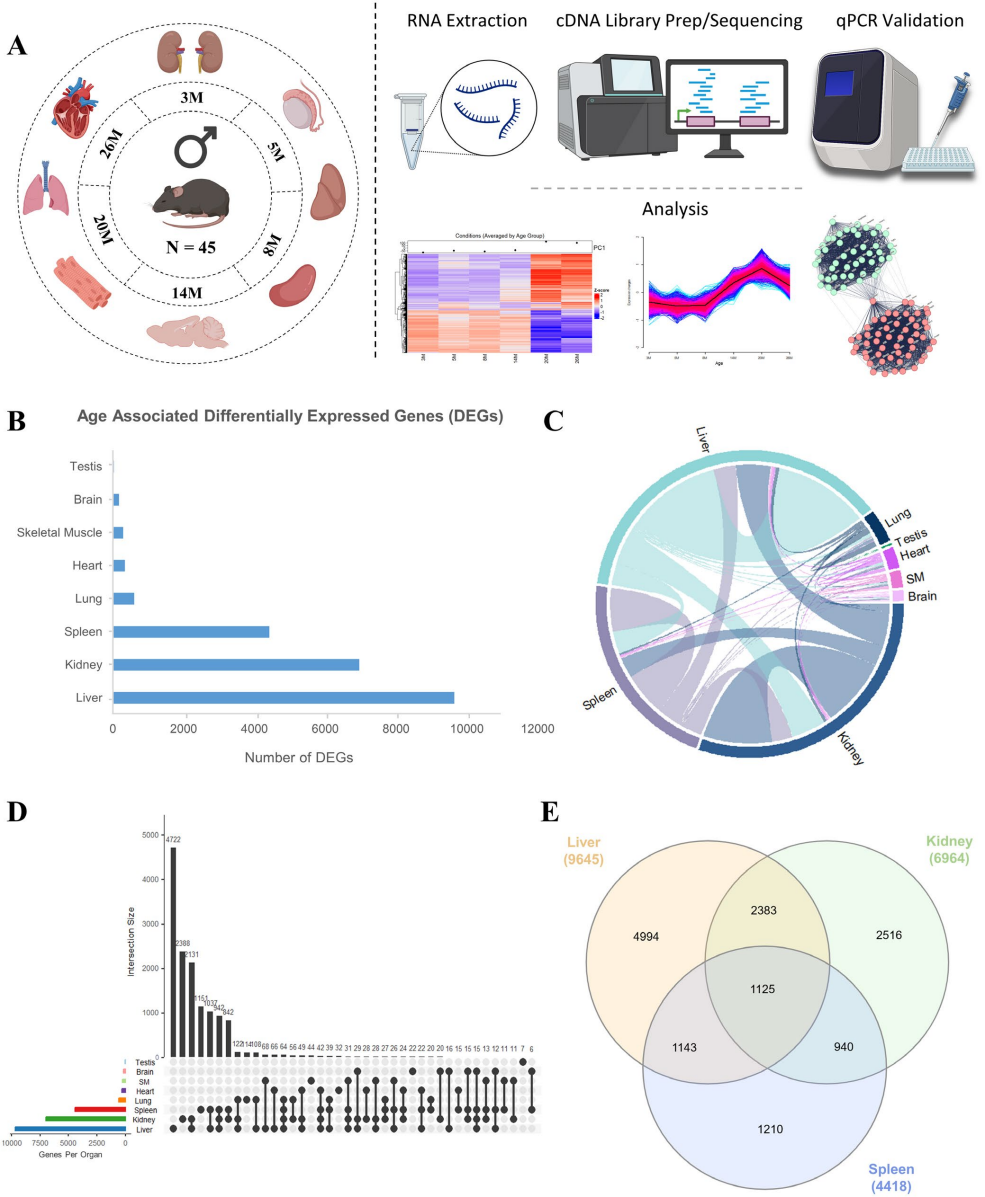
Experimental design and distribution of differentially expressed genes (DEGs) across organs. (A) Schematic representation of the experimental workflow. Male C57BL/6J mice at six different age points (3, 5, 8, 14, 20, and 26 M) were used to examine age‐related gene expression changes across multiple organs (liver, kidney, spleen, lung, skeletal muscle, heart, brain, and testis) from a single experiment. Biological replicates were used in the study for each age group, with for instance: 7 (3 and 20 months), 9 (5 and 14 months), 8 (8 months), and 5 (26 months). The workflow includes tissue collection, mRNA extraction, cDNA preparation and sequencing, qPCR validation and data analysis. Data processing steps included clustering of DEGs, trajectory analysis, and network analysis for the identification of age‐associated gene hubs. Figure generated using BioRender.com. (B) Summary of the total DEGs in each organ. (C) Circos plot illustrating shared and unique DEGs across different organs highlighting the overlap and distinct gene sets associated with aging in each organ. (D) UpSet plot showing intersections of DEGs among the eight assessed organs, with bar heights representing the number of genes shared across various organ combinations. (E) Venn diagram of shared DEGs across the liver, kidney, and spleen. M, months; SM, skeletal muscle. Source data are available online for this figure.

We investigated transcriptomic changes in 8 organs (brain, heart, kidney, liver, lung, skeletal muscle, spleen and testis) from male C57BL/6J mice at 6 age points (3, 5, 8, 14, 20, and 26 months), using bulk RNA sequencing (Figure 1A). To identify differentially expressed genes, we employed a likelihood ratio test (LRT) using a reduced model across all age groups, enabling robust detection of age‐related global gene expression shifts in multiple organs (Bullard et al. 2010) (Figure 1A and Table S1). Furthermore, we evaluated gene expression trajectories of identified DEGs (Figure 1B) using heatmaps and Mfuzz, respectively. To gain insight into age‐associated gene expression changes across mouse organs, we evaluated the overlap of DEGs using set‐based and network visualization approaches (Figure 1C,D). Our analyses revealed a wide range of differential gene expression (DEG) patterns across organs, reflecting the heterogeneous impact of aging on tissue transcriptomes. The liver, kidney, spleen and lung displayed the most extensive transcriptional alterations, with 9645, 6964, 4418 and 604 differentially expressed genes (DEGs), respectively (Figure 1D and Table S2). In contrast, the heart, skeletal muscle, brain and testis exhibited fewer age‐associated DEGs (342, 290, 176 and 29, respectively), suggesting a more limited transcriptomic response to aging in these tissues (Figure 1D and Table S2). Notably, 1125 DEGs were shared between liver, kidney, and spleen (Figure 1E), highlighting a core set of genes underpinning aging‐related remodeling in these organs.

### 2.2 Gene Expression Analyses Across Ages and Organs Show That Aging‐Related Changes Vary in Timing and Magnitude

**FIGURE 2:**
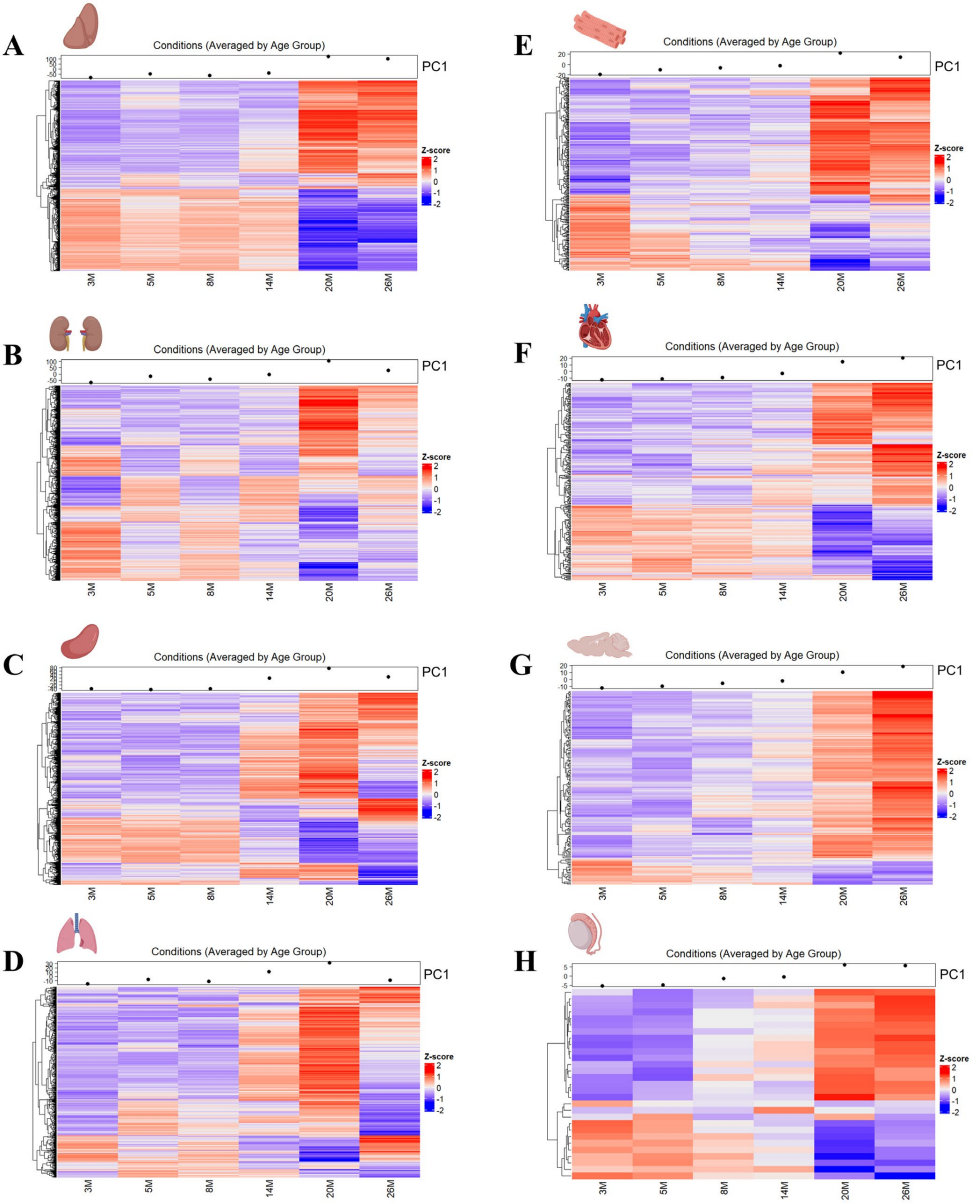
Differential expression profiles featuring age‐associated gene expression changes in liver, kidney, spleen, lung, skeletal muscle, heart, brain, and testis. (A–H) Heatmaps of differentially expressed genes (DEGs) across six age groups (3 M, 5 M, 8 M, 14 M, 20 M, and 26 M) for each organ: Liver (A), kidney (B), spleen (C), lung (D), skeletal muscle (E), heart (F), brain (G) and testis (H). Genes (rows) are clustered (Euclidean) based on similarity in expression patterns across ages (columns). The color gradient represents the z‐scored expression levels, with red indicating upregulation and blue indicating downregulation relative to each gene's mean expression across of all age groups. The top dot plots display the principal component 1 (PC1) trajectory of the z‐scored expression profiles, highlighting shifts in gene expression across ages. The variance explained by PC1 is for the liver (88.7%), kidney (58%), spleen (57%), lung (60.6%), skeletal muscle (70.3%), heart (75.6%), brain (81.8%), and testis (84.5%). Source data are available online for this figure.

To further explore the molecular dynamics of aging, we generated heatmaps of DEGs and conducted Principal Component Analysis (PCA) across 8 organs (Figure 2A–H), focusing on the first principal component (PC1) to capture age‐related transcriptomic trends (Table S3). These visualizations provided a comprehensive view of gene expression trajectories with age, revealing key differences in the onset, rate, and magnitude of transcriptional changes across the different organs.

The liver displayed a distinct pattern of resistance to early aging‐related changes, maintaining stable gene expression from 3 to 14 months before a sharp shift occurred at 20 months and stabilized by 26 months (Figure 2A). In contrast, the kidney, spleen, and lung exhibited earlier transcriptomic alterations. These organs remained stable until 8 months but underwent progressive changes peaking at 20 months, with partial reversal by 26 months (Figure 2B–D). Notably, the spleen and lung displayed a more rapid onset compared to the gradual progression observed in the kidney during this period. The skeletal muscle showed an earlier onset of transcriptional alterations, with shifts beginning as early as 8 months after an initial period of stability (3–5 months). These changes peaked at 20 months and were maintained at similar levels through 26 months, indicating greater susceptibility to early age‐related alterations (Figure 2E). A similar pattern of age‐related transcriptomic changes with the kidney, spleen, and lung was observed in the heart until 20 months, followed by a continued increase in changes even at 26 months (Figure 2F). Similarly, to the liver, the brain remained largely stable until 14 months but in addition exhibited increased transcriptomic changes at 20 and 26 months, as observed in the heart (Figure 2G). Interestingly, the persistent increase in transcriptomic changes in both the heart and brain at 26 months suggests sustained late‐life transcriptional remodeling in both organs. The testis displayed a similar profile of transcriptomic changes as observed in the skeletal muscle (Figure 2H). Collectively, aging‐associated alterations remain relatively stable across all assessed organs from 3 to 8 months, followed by a pronounced transition thereafter. Notably, the testis and skeletal muscle deviate from this pattern, displaying distinct early, mid, and late‐phase changes across the lifespan.

Importantly, the overall magnitude of transcriptional changes as indicated by PCA also varied significantly between organs, ranging from very low (testis), low (brain and heart), moderate (lung and skeletal muscle), to high (kidney, liver and spleen) (Figure 2A–H). These findings provide a nuanced understanding of the temporal and organ‐specific dynamics of aging, emphasizing shared and unique patterns in the onset and magnitude of transcriptomic changes.

### 2.3 Temporal Trajectories and Functional Characterization of Age‐Associated Genes Across Organs and Life Stages

**FIGURE 3:**
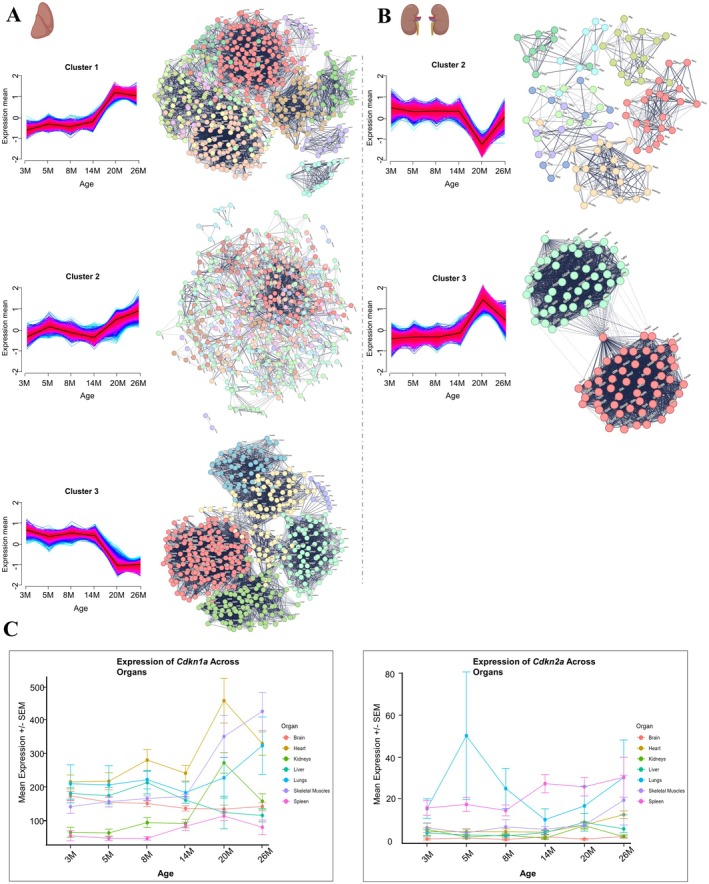
Age‐associated gene expression trajectories and hub gene network analyses in liver and kidney. (A–B) Mfuzz clustering of z‐scored expression profiles of differentially expressed genes (DEGs) reveals distinct temporal expression trajectories across aging in liver (A) and kidney (B). Each subpanel shows cluster‐specific expression patterns over time, with average trajectories highlighted. Next to each cluster plot, gene interaction networks of top hub genes are displayed. Hub genes were identified using maximal clique centrality (MCC) scores via CytoHubba and visualized in STRING based on STRING protein–protein interaction data. Nodes represent genes, and edges represent functional associations, highlighting highly connected nodes (“hubs”), with potential roles in coordinating functional networks. For network visualization, the following numbers of top hub genes per cluster were used: Liver‐top 500 genes and kidney‐top 100 genes. (C) Line plots showing the expression dynamics of senescence markers Cdkn1a and Cdkn2a across the six age groups in each organ. Lines are color‐coded by organ, illustrating distinct, organ‐specific temporal expression profiles of these key genes during aging.

To unravel the complex transcriptomic landscape of aging across different organs, we applied a multilayered analytical approach. We first performed time series clustering of DEGs using Mfuzz, focusing on dynamic expression patterns over the lifespan (see “Materials and methods”, section 4.4). Each organ exhibited distinct cluster‐specific gene expression trajectories, reflecting diverse molecular changes. The most prominent clusters and their corresponding gene hub interaction networks are shown for the liver (Figure 3A), kidney (Figure 3B), spleen (Figure S1), lung (Figure S2A), and skeletal muscle (Figure S2B).

In the liver, three main gene expression patterns emerged, with clusters 1 and 3 showing the strongest signals. Cluster 1 was characterized by a relatively steady gene expression from 3 to 14 months, followed by a pronounced rise from 14 to 20 months, which then stabilized between 20 and 26 months (Figure 3A, left). Gene Ontology (GO) enrichment analysis revealed that this cluster was enriched for pathways related to metabolic and catabolic processes as well as transmembrane transport (Figure 3A, right and Table S4). Cluster 2 displayed a biphasic trajectory, marked by a mostly stable gene expression from 3 to 5 months, a decline through 14 months, and a subsequent rise from 20 months onward (Figure 3A, left). Surprisingly, genes in this cluster were enriched in terms associated with “modulation of chemical synaptic transmission” and “synaptic signaling,” typically linked to neuronal function but possibly reflecting hepatic involvement in neuroendocrine regulation, inflammation, or vesicular trafficking (Roskams et al. 2004) (Figure 3A, right and Table S4). Cluster 3 showed a slight decline between 3 and 5 months, remained relatively stable until 14 months, and then sharply decreased at 20 months, maintaining this lower level through 26 months (Figure 3A, left). This cluster was enriched in “oxidative phosphorylation,” “respiration,” and “energy production,” consistent with declining mitochondrial function and bioenergetic stress in aging hepatocytes (Figure 3A, right and Table S4).

In the kidney, clusters 2 and 3 dominated the transcriptional landscape. Both clusters remained relatively stable from 3 to 14 months. Cluster 2 then exhibited a sharp downregulation between 14 and 20 months, followed by a partial recovery by 26 months (Figure 3B, left). GO enrichment analysis for this cluster highlighted metabolic and catabolic processes (Figure 3B, right and Table S4). Conversely, cluster 3 showed a marked increase in gene expression from 14 to 20 months, followed by a decline by 26 months (Figure 3b, left). This cluster was enriched in genes implicated in immune system processes, stress responses, as well as innate and adaptive immunity, reinforcing the role of inflammation and immune engagement in aging kidney pathology (Figure 3B, right and Table S4).

The spleen displayed a complex aging trajectory with five prominent clusters (clusters 1–5) showing distinct temporal patterns. Most clusters remained stable from 3 to 8 months, except for cluster 3, which maintained stability until 14 months. Cluster 1 exhibited a gradual increase in gene expression between 8 and 20 months, followed by a slight decline by 26 months (Figure S1, left). GO enrichment linked this cluster to processes such as “regulation of multicellular organismal processes,” “response to external stimulus,” and “cell migration,” suggesting active remodeling of splenic function during aging (Figure S1, right and Table S4). Cluster 2 showed decreased gene expression between 8 and 20 months, stabilizing by 26 months (Figure S1, left). The GO terms enriched in this cluster included lymphocyte‐proliferation, expansion and activation, “adaptive immune system process,” “positive regulation of cytokine production,” which may suggest reduced lymphopoiesis and impaired adaptive immunity as hallmark features of the aging spleen (Figure S1, right and Table S4). In contrast, cluster 3 exhibited persistent upregulation beginning at 14 months. This cluster was enriched for terms such as “mitotic nuclear division,” “G2/M transition,” and “DNA replication,” consistent with sustained activation of the core mitotic machinery reflecting active cell cycle progression, suggestive of heightened proliferative activity potentially indicative of maladaptive proliferation (Figure S1, right and Table S4). Cluster 4 displayed an increase in gene expression between 8 and 20 months, followed by a sharp decline by 26 months (Figure S1, left). The pathways enriched in this cluster were primarily associated with “system development” and “anatomical structure morphogenesis,” pointing to potential adaptive or maladaptive structural remodeling within the aging spleen (Figure S1, right and Table S4). Finally, cluster 5 showed decreased gene expression between 8 and 20 months, followed by a sharp increase by 26 months (Figure S1, left). GO analysis revealed enrichment in pathways related to “cytoplasmic translation,” “translation,” and “ribosome biogenesis,” implicating dysregulated protein synthesis and impaired proteostasis during late‐life spleen aging (Figure S1, right and Table S4).

In the lung, two prominent gene expression clusters emerged, each displaying distinct temporal patterns. Cluster 1 showed stable gene expression between 3 and 8 months, followed by a marked increase from 8 to 20 months, and a partial decline thereafter (Figure S2A, left). GO analysis revealed that this cluster was significantly associated with immune system processes and adaptive immune activation, suggesting a mid‐life surge in immune activity (Figure S2A, right and Table S4). In contrast, cluster 3 exhibited a modest increase in gene expression between 3 and 5 months, followed by sustained stability through 20 months, and a decline thereafter (Figure S2A, left). Genes within this cluster were enriched for “ion and amino acid transport” as well as “synapse assembly and organization,” indicating an aging‐associated change in lung homeostasis and intercellular communication (Figure S2A, right and Table S4).

For skeletal muscle, clusters 1 and 2 represented the dominant trajectories. Cluster 1 displayed a relatively stable gene expression from 3 to 14 months, followed by a sharp decline between 14 and 20 months, which stabilized by 26 months (Figure S2B, left). GO analysis of this cluster highlighted enrichment in “collagen fibril organization” and “extracellular matrix organization” suggestive of progressive structural deterioration and compromised integrity in aging skeletal muscle (Figure S2B, right and Table S4). In contrast, cluster 2 maintained relatively stable expression levels from 3 to 8 months, followed by a gradual increase between 8 and 14 months, which became more pronounced from 14 months onward (Figure S2B, left). Functional annotation linked this cluster to pathways involved in “negative regulation of T cell activation” as well as “intrinsic apoptotic signaling pathway by p53 class mediator” pointing to immune modulation and apoptotic processes contributing to skeletal muscle aging (Figure S2B, right and Table S4).

In the heart, trajectory analysis revealed 2 dominant gene expression clusters, each capturing distinct aging‐associated transcriptional programs (Figure S2C). Cluster 1 followed a biphasic pattern, with gene expression gradually decreasing until 14 months followed by a sharp decline at 20 months and late‐life upregulation. Functional enrichment linked this cluster predominantly to “gas transport,” “oxygen transport,” “carbon dioxide transport,” and “erythrocyte development,” indicating a possible mid‐life decline and late compensatory response in oxygen handling and erythropoietic capacity (Figure S2C and Table S4). Cluster 3 of the heart was characterized by a gradual and sustained increase in gene expression across the lifespan. GO enrichment analysis of this cluster highlighted significant involvement in “immune system processes” and “response to external stimuli” reflecting progressive activation of inflammatory pathways during cardiac aging (Figure S2C and Table S4).

Finally, in the brain, cluster 2, which was the most prominent, exhibited a gradual gene expression increase from 3 to 14 months, followed by a rapid increase that continued until 26 months (Figure S2D). Functional annotation of the cluster indicated overrepresentation of genes encoding “immune response” and “response to stress” pathways (Table S4).

### 2.4 Cellular Senescence Marker Dynamics Across Organs

Given the pivotal role of cellular senescence in driving age‐associated tissue remodeling and functional decline (Kumar et al. 2020), we next examined the expression profiles of two canonical senescence and aging markers, *Cdkn1a* and *Cdkn2a* (Khosla et al. 2020), across all organs (excluding the testis where both markers were minimally expressed). Both genes exhibited dynamic, organ‐specific expression changes with age, reinforcing their value as molecular indicators of senescence (Figure 3C). *Cdkn1a* expression remained relatively stable in most organs during early to mid‐life but increased markedly at late ages, particularly in the kidney, lungs, heart, and skeletal muscle, which showed patterns consistent with progressive senescence‐driven changes in these tissues. The liver displayed a slight decrease in *Cdkn1a* expression at late ages. In contrast, *Cdkn2a* expression demonstrated a more modest and variable pattern where most organs maintained relatively low and stable levels throughout life, except for the heart, spleen, and skeletal muscle, which displayed increased levels at late ages.

To explore how senescence‐related processes evolve with age across different organs, we assessed the activity of a senescence gene module (“SenMyo,” see Table S5A) (Saul et al. 2022) previously implicated in cellular senescence. Using gene set variation analysis GSVA (Hänzelmann et al. 2013), we computed a SenMyo module score (Table S5B) for each sample based on the full transcriptomic matrix per organ. To formally test cross‐organ heterogeneity of senescence activity, we fit a linear mixed model (LMM) with Organ as a fixed effect and MouseID as a random intercept to account for repeated measures within individuals. The Age×Organ interaction was significant, indicating that the age effect on SenMyo differs by organ. We next extracted organ‐specific age slopes (GSVA units/month ± SE; 95% CI) revealing a clear gradient of senescence progression across tissues. The kidney exhibited the steepest increase (0.0329 ± 0.0035; 95% CI: 0.0260–0.0399), followed by the spleen (0.0178 ± 0.0035), heart (0.0132 ± 0.0035), and brain (0.0120 ± 0.0035). A more moderate increase was observed in skeletal muscle (0.0070 ± 0.0035), whereas lung (0.00313 ± 0.00354) and liver (−0.00184 ± 0.00354) showed minimal to no age‐related trend (Figure S3A,B and Table S5C). Consistent with these slopes, SenMyo activity increased early in the kidney (≈5 months) and peaked by 20 months; the spleen rose strongly beginning at ~8 months; the heart (from ~8 months) and brain (from ~14 months) increased more gradually; and skeletal muscle showed a later shift (≈20 months) (Figure S3B).

To capture potential non‐linear age trajectories, we fit a generalized additive model (GAM; REML) for each organ, modeling SenMyo score as a smooth function of age. The most prominent non‐linear increase was observed in kidney (*p* < 6.9 × 10^−11^; Spearman *ρ* = 0.80; (Δ26 M—3 M) = 0.62), followed by brain (*p* = 1.8 × 10^−6^; *ρ* = 0.60; Δ = 0.26), spleen (*p* = 2.4 × 10^−4^; *ρ* = 0.50; Δ = 0.175), and heart (*p* = 1.5 × 10^−3^; *ρ* = 0.45; Δ = 0.296). Skeletal muscle showed a milder but significant pattern (*p* = 0.035; *ρ* = 0.18), while the lung and liver were not significant (*p* > 0.25), consistent with their flat (lung) or slightly negative (liver) LMM slopes (Figure S3C and Table S5D). These analyses reveal that senescence module activity increases with age in an organ‐specific and often non‐linear manner, with the kidney showing the strongest signal.

To identify key genes driving these organ‐specific senescence patterns, we integrated two criteria: (i) a significant age‐gene association within each organ (Spearman FDR < 0.05), and (ii) strong coherence with the SenMyo score (Correlation between gene expression and GSVA score), with consistent directionality. Genes were ranked using a composite score: |R gene module| × |*ρ*_age| × −log10(FDR). The top 20 age‐upregulated genes per organ are visualized in Figure S3D (see Table S5E for full list). Across organs, the top age‐upregulated SenMyo genes converged on inflammatory/SASP signaling (*Tnfrsf1a/b, Ccl7/8, Cxcl1/2/12/16, Il1b*), ECM remodeling (*Mmp2/3/12/14*), and complement (*C3*), indicating shared senescence‐associated inflammatory and matrix processes. Notably, the kidney showed a fibrotic/inflammatory bias (*Spp1, Mmps, Icam1, Il1b, C3*), the spleen emphasized ECM/complement with growth‐factor signaling (*Mmps, C3, Serpine1*, *Plat/Plau, Igf/Hgf* axis), the heart favored inflammatory SASP with EGFR‐axis stress/growth factors (*Ccl8, Cxcl16, Mmp3, Ereg, Gdf15*), and the brain exhibited microglial/inflammatory chemokines with complement and proteases (*Mmp12, C3, Cxcl12/16, Csf1, Icam1, Axl*) (Figure S3D and Table S5E). These patterns support heterogeneous, organ‐biased representation of senescence markers across organs.

We further examined how these SenMyo trajectories relate to canonical senescence markers *Cdkn1a* and *Cdkn2a*. Correlating SenMyo scores with *Cdkn1a/2a* expression across age revealed strong positive coupling in the spleen (both markers), kidney (both markers), and liver (*Cdkn1a* only, albeit decreasing with age). In contrast, coupling was weaker or inconsistent in the brain and skeletal muscle (Figure S3E and Table S5F). Finally, to map gene‐level relationships, we correlated the expression of each SenMyo gene with *Cdkn1a* and *Cdkn2a* within each organ (Figure S3F and Table S5G). Figure S3F and Table S5 highlight subsets of SenMyo genes co‐expressed with these canonical markers which weren't completely similar across organs, reinforcing the idea that distinct senescence gene subsets dominate the aging response in each organ.

### 2.5 Network Analysis Identifies Central Gene Hubs Strongly Associated With Organ‐Specific Aging Signatures

To further dissect the molecular drivers underlying the observed transcriptomic alterations described above, we performed a network analysis on DEGs across all organs except the testis (excluded due to its limited number of DEGs). Using Cytoscape and Cytohubba (Chin et al. 2014), we identified the most highly connected gene hubs based on Maximal Clique Centrality (MCC), offering insight into potential key regulators orchestrating age‐associated gene expression shifts within each organ‐specific cluster.

In the liver, network analysis revealed distinct aging‐related signatures predominantly in clusters 1 and 3 (Figure 3A, right). Cluster 1 featured the progressive upregulation of genes involved in metabolic and detoxification pathways, including cytochrome P450 (CYP) enzymes (e.g., *Cyp1a2, Cyp2c70*, and *Cyp2e1*), apolipoproteins (*Apoa1, Apoa2, Apob*, and *Apoe*), and lipid transport regulators (*Fabp1, Ldlr*, and *Lrp1*). Additional highly connected hubs included genes encoding acute‐phase proteins (*Crp, Hpx*, and *Serpina1a*), glutathione‐S‐transferases (*Gsta2* and *Gstp1*), and core metabolic regulators (*Alb*, *Pck1*, and *Ttr*) (Figure 3A, right and Table S6).

Cluster 2 revealed an intriguing enrichment of genes typically associated with neuronal signaling. Genes encoding synaptic proteins, including *Cacng2, Dlg4, Gria1, Gria2, Grin1*, and *Grin2b*, as well as key components of AMPA and NMDA receptor signaling, were progressively upregulated. Notably, gene hubs like *Drd1*, *Gria1‐4*, *Grin1*, *Grin2b*, *Grm5*, and GABAα receptor genes were also highly connected, mirroring gene expression patterns observed in liver fibrosis, cirrhosis, and hepatocellular carcinoma (HCC) (Stepanov et al. 2022; Wang et al. 2017; Zeng et al. 2023). Furthermore, this cluster showed upregulation of adrenergic receptors (*Adra1a*, *Adra1b*, *Adra2b*, and *Adrb3*) and genes related to vesicular trafficking (*Bet1* and *Vamp8*) and exocytosis (*Snap25* and *Stx1b*), highlighting a potential neuroendocrine and vesicular component to hepatic aging.

The most connected gene hubs in cluster 3 (Figure 3A, right and Table S6) indicated downregulation of genes encoding the mitochondrial ETC (e.g., *Cox4i1, Cox5a, Cox5b, Ndufa2, Ndufs2, Ndufs3*, and *Uqcrc1*), ATP synthase subunits (e.g., *Atp5f1c* and *Atp5mc3*), and TCA cycle enzymes (e.g., *Cs* and *Idh3a*). Additionally, mitochondrial ribosomal genes (*Mrpl13, Mrpl19, Mrpl30, Mrpl41*, and *Mrpl47*), antioxidant genes (*Prdx3* and *Sod2*), mitochondrial biogenesis regulators (*Ppargc1a* and *Tfam*), and a cAMP signaling associated gene (*Prkar1a*) were also downregulated in this cluster.

In the kidney, we further interrogated clusters 2 and 3, previously identified as dominant in the trajectory analysis (Figure 3B). Cluster 2 contained gene hubs enriched for regulators of metabolic and oxidative stress‐related processes. Identified central hubs included *Cbs* (transsulfuration pathway), *Idh2* (TCA cycle and energy production), *Ppargc1a* (mitochondrial biogenesis regulator), *Sirt3* (critical mitochondrial regulator), as well as *Sod2* and *Shmt2* (redox balance). The top gene hubs in cluster 3 (Figure 3B, right) were primarily associated with inflammatory signaling, immune activation, and fibrosis‐related genes, all of which are strongly upregulated at 20 months. Among these, *Ccr5, Cd86, Icam1, Il6, Il10ra, Ptprc, Stat3, Tgfβ1, Tlr4, Tnf*, and *Vcam1* were found to be the key regulators (Figure 3B, right and Table S6). Additionally, senescence‐associated genes such as *Cdkn1a, Cdkn2a, Lag3*, and *Serpine1* were upregulated.

In the spleen, gene hubs were identified from clusters 1–5 (Figure S1, left). Cluster 1 exhibits a dynamic upregulation of ECM‐associated genes, particularly multiple collagen family members (*Col3a1, Col4a1, Col4a2, Col5a1, Col6a1, Col6a2, Col6a3*, and *Col18a1*) in addition to *Tgfb1*, which peak at 20 months before declining at 26 months (Figure S1, right and Table S6). Cluster 2 exhibits a significant downregulation between 8 and 20 months, predominantly affecting adaptive immune system‐related genes, including *Bcl6, Ccr7, Cd3e, Cd4*, and *Cd28* (Figure S1, right and Table S6). GO enrichment analysis further supports this trend, highlighting processes such as lymphocyte activation, leukocyte activation, and immune system regulation. Central hub genes such as *Card11, Gata3, Lef1, Mef2c*, and *Runx1* (Figure S1, right and Table S6), crucial for T‐cell and B‐cell differentiation, maturation and activation (Hsu et al. 2016; Pomerantz et al. 2002; Wang et al. 2011; Wilker et al. 2008; Xing et al. 2016), also showed marked downregulation (Figure S1, right and Table S6). In contrast, cluster 3 exhibits a progressive upregulation from 14 months onward, characterized by a strong enrichment in cell cycle regulators with the dominance of hub genes including *Aurkb, Bub1b, Ccnb1, Cdc20, Chek1*, and *Plk1* (Figure S1, right and Table S6). Similarly to cluster 1, cluster 4 was dominated by genes linked to ECM remodeling, fibrosis, and stromal activation, with expression increasing steadily from 8 to 20 months yet with a more pronounced decline at 26 months. The top gene hubs in the cluster include: *Col1a1, Pdgfrb*, and *Tgfb2* (central regulators of fibrotic processes), *Col1a2* and *Col3a1* (other ECM components), as well as *Admts5* and *Serpine1* (matrix regulators) (Figure S1, right and Table S6). Finally, Cluster 5 in the aging spleen is characterized by a significant downregulation of several ribosomal gene families (*Rpl, Rplp*, and *Rps*), which encode essential proteins for translation (Figure S1, right and Table S6).

For the lung, skeletal muscle, heart, and brain, we had fewer genes per cluster compared to the above‐mentioned organs and therefore used the entire gene lists instead of gene hubs for our investigation. The 29 age‐associated DEGs determined in the testis were too few to perform network analysis or functional annotation. In the lung, genes related to immune response (*Cd22, Cd3g, Ctla4, Cybb, Hck, Ikzf1, Il7, Itgam, Itk, Ikzf1*, and *Lck*), inflammation (e.g., *Alox5* and *Nlrp12*), and chemokine signaling (*Ccr5, Cx3cr1*, and *Cxcl9*) were enriched in cluster 1 (Figure S2A, right and Table S6). Cluster 2 of the lung was enriched in genes related to peroxisomal oxidation (*Hao1* and *Hao2*) and cytochrome P450 detoxification (*Cyp1a2, Cyp2a12*, and *Cyp2c54*). In addition, we also identified urate metabolism (*Slc17a1*) and synaptic neurotransmission (*Slc17a8*) associated genes in the cluster (Figure S2A, right and Table S6).

Cluster 1 of the skeletal muscle was enriched in genes encoding collagens (*Col1a1, Col1a2, Col6a1*, and *Col6a2*) and metabolic regulators (*Amd1, Amd2, Odc1, Pdk4, Ucp3*, and *Smox*) (Figure S2B, right). In cluster 2, the identified genes consisted of inflammatory and immune‐related genes (*Ccl8, Il1b*, and *Slfn4*), stress response and metabolic regulators (*Klf5, Gpx3*, and *Osmr*), ECM and muscle homeostasis genes (*Ctss* and *Lgmn*), as well as the senescence marker *Cdkn1a* (Figure S2B, right). In the heart, both cluster 1 and cluster 3 (Figure S2C) consisted of genes related to innate immune responses (*Aim2, Ccr1, Ccr2*, and *Tlr4*), proinflammatory mediators (*Ccl8, Cxcl2, Cxcl5, Cxcl13*, and *Cxcl16*), adaptive and innate immune modulatory markers (*Fcgr2b*, *H2‐Q7, Nlrc5*, and *Ptprc* (*Cd45*)), as well as *Cdkn1a, Gdf15, Lox, Postn, Mmp3*, and *Serpina3n*. Genes related to oxygen transport (*Hbb‐bs*), heme biosynthesis (*Alas2*), and extracellular matrix regulation (*Mgp*) were identified in cluster 2 of the heart (Figure S2C). Finally, cluster 2 was the most prominent in the brain and comprised immune‐related genes (e.g., *Ccl3, Ccl4, Cd74, Clec7a, Cst7, Cybb, Fcgr2b, Ptprc*, and *Tlr2*) (Figure S2D). In addition, we identified *C3* and *C4b*, both of which were upregulated, as well as *Nptx2*, which was downregulated.

### 2.6 Structural Remodeling, Immune Response, Inflammation, and Vascular Dysfunction Are Shared Features of Aging Across Organs

**FIGURE 4:**
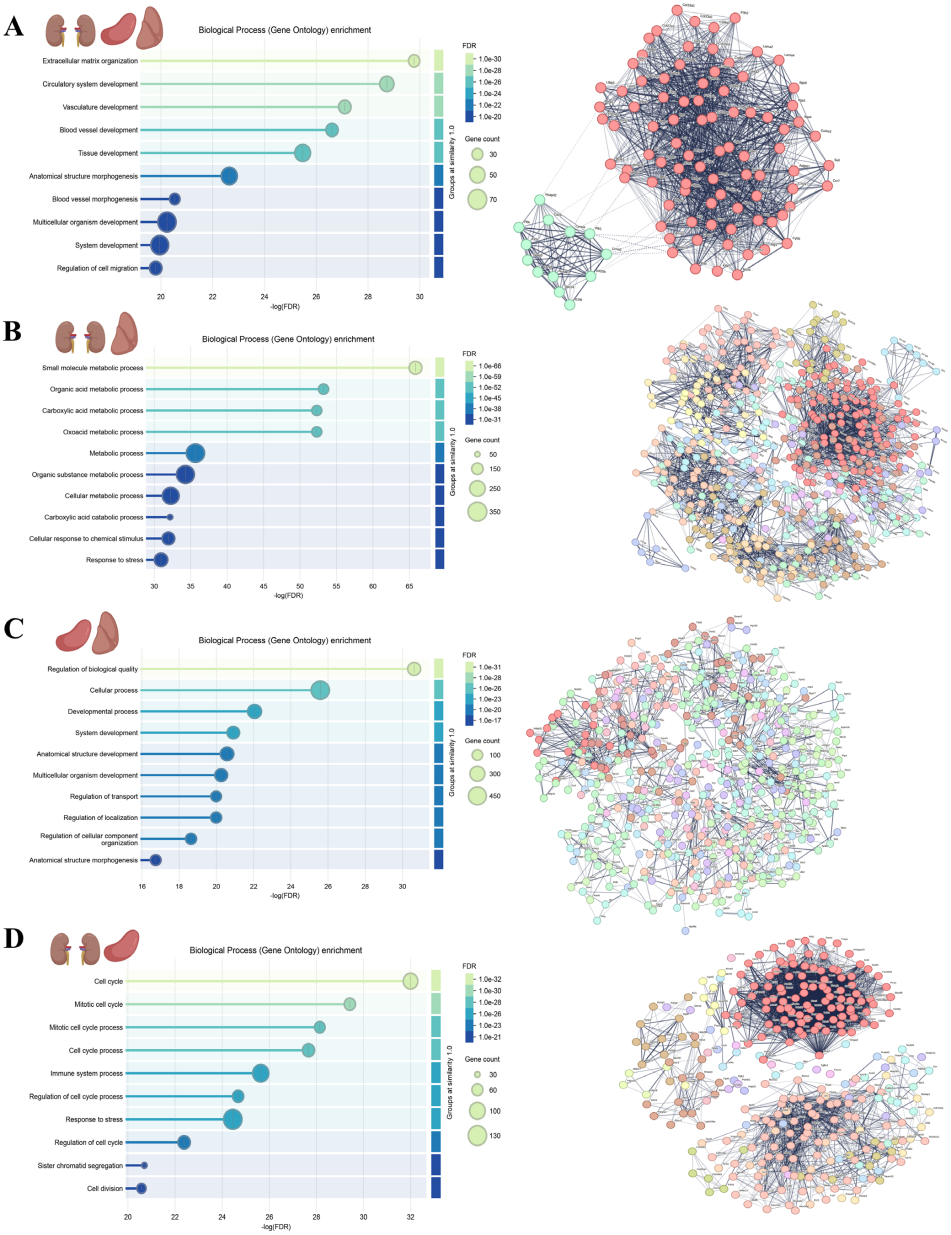
Shared gene network and functional enrichment analysis across organ pairs/groups. (A–D) Gene Ontology (GO) biological process enrichment and STRING interaction networks of top hub genes shared between organ pairs or groups: Kidney‐spleen‐liver (A), kidney‐liver (B), spleen‐liver (C), and kidney‐spleen (D). Left panels: GO enrichment dot plots for biological processes associated with the shared hub genes. The x‐axis represents the significance of enrichment as –log_10_(FDR). Dot size reflects the number of genes contributing to each term, while color indicates the FDR value. Right panels: STRING protein–protein interaction (PPI) networks of the top hub genes identified using CytoHubba's maximal clique centrality (MCC) ranking in Cytoscape. Nodes represent genes, and edges represent functional associations, highlighting highly connected nodes (“hubs”), with potential roles in coordinating functional networks. Source data are available online for this figure.

**FIGURE 5:**
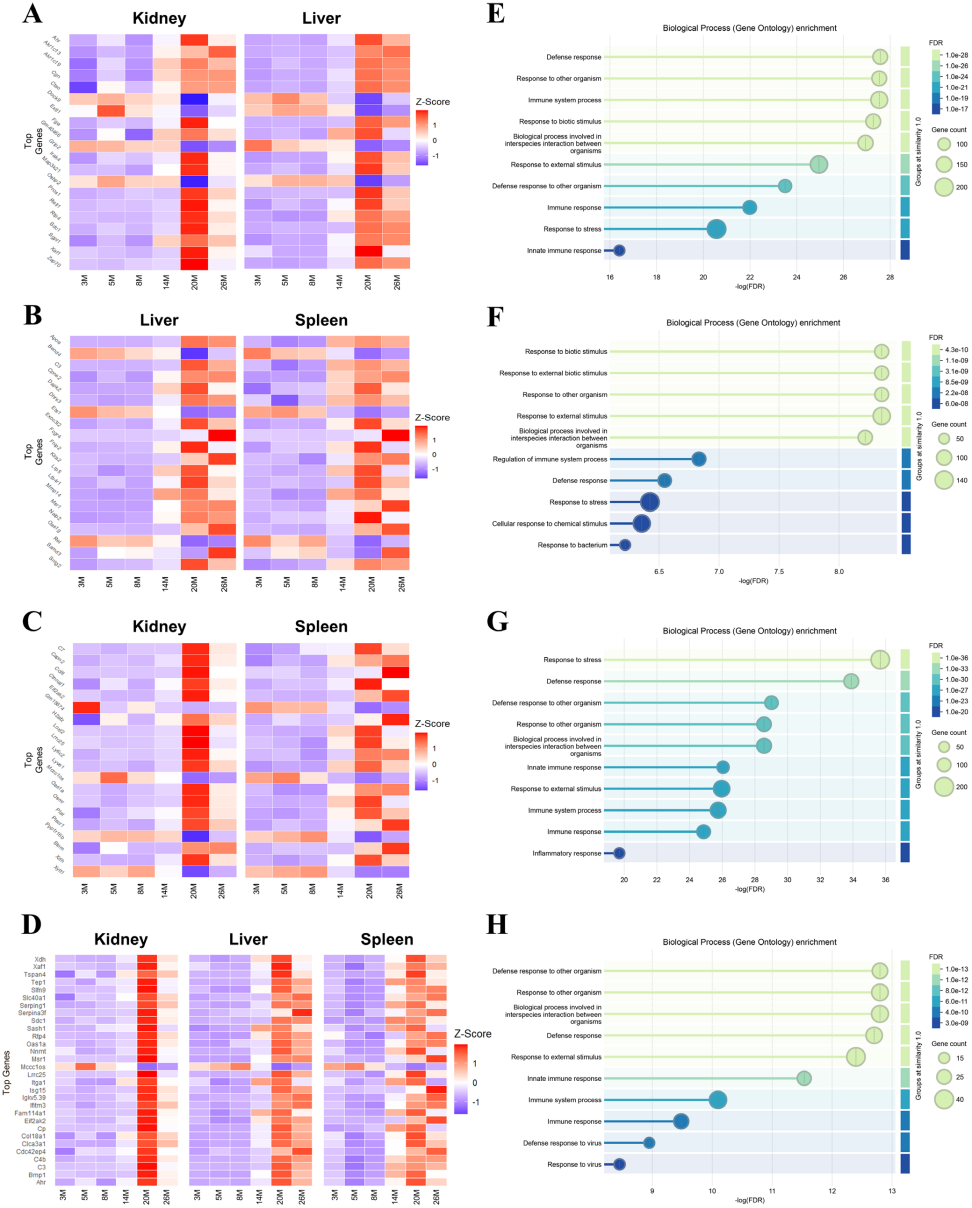
Cross‐organ correlation of age‐associated gene trajectories and functional enrichment of positively correlated genes. (A–D) Heatmaps showing the expression trajectories of the top 20 positively correlated genes (Spearman's *ρ* > 0.5) shared between paired organs: Kidney‐liver (A), liver‐spleen (B), and kidney‐spleen (C). The top 30 positively correlated genes (Spearman's *ρ* ≥ 0.5) shared across kidney, liver, and spleen are shown in (D). Columns represent age groups (3, 5, 8, 14, 20, and 26 M), and rows represent genes. Color scale reflects z‐scored expression levels (red: Upregulation, blue: Downregulation) relative to each gene's mean expression across ages. (E, F) Gene Ontology (GO) biological process enrichment analysis of the positively correlated genes (Spearman's *ρ* > 0.5) for each organ pair: Kidney‐liver (E), liver‐spleen (F), kidney‐spleen (G), and the triad kidney‐liver‐spleen (H). The x‐axis shows –log_10_(FDR) values indicating enrichment significance. Bubble size corresponds to gene count, and color reflects FDR values, with lighter shades indicating higher significance. Enriched terms highlight shared functional pathways underlying coordinated age‐related changes across organs. GO analysis was done via STRING. Source data are available online for this figure.

Toward unraveling common central components of aging signatures across organs, we performed network analyses and focused on age‐associated differentially expressed genes shared across organ pairs and groups. Most shared genes were found between the kidney, spleen, liver, and lung, with 3508, 2065, and 2268 DEGs shared between the organ pairs: kidney/liver, kidney/spleen, and liver/spleen, respectively (Table S2). 1125 and 209 DEGs were shared between the organ triads: kidney/liver/spleen and kidney/liver/lung, respectively. Only 81 DEGs were shared among 4 organs, namely kidney, spleen, liver, and lung (Table S2). As such, we focused on the three organ pairs and a triad with the most shared DEGs to highlight common aging‐related features across the organs (Figures 4A–D and 5A–H).

Gene ontology analysis of the shared aging‐associated genes between the kidney, liver, and spleen revealed predominant enrichment in ECM organization, circulatory system development and vasculature development pathways (Figure 4A). Key genes contributing to these pathways include multiple collagens (*Col1a1, Col1a2, Col3a1, Col4a1*, and *Col6a1*), fibrosis regulators (*Ccn2, Fbn1, Lox*, and *Thbs1*), and matrix metalloproteinases (*Mmp2* and *Mmp9*) (Table S7). In addition, genes encoding vascular growth factors (*Edn1, Flt1, Kdr, Pdgfb*, and *Vwf*) were also identified in this category (Table S7).

Next, we identified the top gene hubs unique to the organ pairs kidney/liver (Figure 4B), spleen/liver (Figure 4C), kidney/spleen (Figure 4D), with the aim of zooming in on the most dominant shared age‐related processes between each organ pair. Functional annotations of the top 500 gene hubs unique to the kidney/liver (Figure 4B) revealed a strong dominance of metabolic processes, particularly small molecules, organic acid, and carboxylic acid metabolism. Key genes driving these pathways include central metabolic regulators, enzymes involved in energy production and fatty acid metabolism (*Acadm, Acox2, Dbt, Ftcd*, and *Gcdh*), as well as amino acid catabolism (*Ahcy, Cbs, Gcdh*, and *Glud1*). The enrichment of cytochrome P450 family members (*Crot, Cyp2d12, Cyp2d26*, and *Cyp51*) further underscores the involvement of xenobiotic and lipid metabolism (Figure 4B and Table S7).

GO analysis of the top 500 gene hubs of shared age‐DEGs between the spleen and liver (Figure 4C) revealed dominant enrichment in biological quality control, cellular processes, and system development pathways. These were driven by key regulators of redox balance and cellular homeostasis, including *Blvrb* and *Gstt1* (Figure 4C and Table S7). Enrichment in system development and anatomical structure remodeling, involving *Egfl7, Esam, Lamb1*, and *Smo*, suggests roles played by vascular, structural, and immune adaptations in this organ pair (Figure 4C and Table S7). Notably, the presence of neuro‐immune and cytoskeletal regulators like *Cacna1c, Jup, Lbr*, and *Rhoc* further supports dynamic structural remodeling (Table S7).

Based on functional annotation of the top 300 gene hubs unique to the kidney‐spleen (Figure 4D), we identified strong enrichment in cell cycle and mitotic processes. This was driven by a large set of cell cycle regulators, including *Aurkb, Bub1b, Brca1, Ccnb1/2, Cdk1*, and *Mki67* (Table S7), suggesting heightened cell division and potential hematopoietic activity. Immune system processes were also significantly enriched, with contributions from *Cd79a, Fcgr1, Il7r, Pparg*, and *Trem2* (Table S7), indicating active immune cell expansion, myeloid activation, and lymphoid lineage engagement. In addition, *Cdkn1a* (p21), a key senescence marker and cell cycle inhibitor, was detected among the shared gene hubs in the kidney and spleen. Together, these findings imply that the spleen‐kidney axis in aging is characterized by a dynamic balance between proliferative drive, immune remodeling, and emerging senescence, reflecting complex tissue responses to chronic stress and damage accumulation.

### 2.7 Innate Immune Response Genes Are Significantly Overrepresented and Show Strong Positive Correlations in Aging Kidney, Liver and Spleen

To identify genes with similar expression profiles across the kidney, liver, and spleen over time, we assessed the trajectory correlation of transcriptomic changes (Table S8) among shared DEGs in the three organs and organ pairs‐kidney and spleen, kidney and liver, as well as liver and spleen‐using Spearman's rank correlation coefficient (Spearman 2010). In addition, we also investigated the expression trends in organ pairs, including kidney and spleen, kidney and liver, as well as liver and spleen (Figure 5A–F). Expression levels of shared differentially expressed genes associated with “defense response” and “innate immune response,” including *C3, C4b, Clec4n, Cybb, Eif2ak2, Ifitm3, Irf7, Lbp, Sdc*, and *Serping1*, were highly correlated in the kidney, liver, and spleen (Figure 5A–F and Table S8). Similarly, the kidney and liver showed high correlation of shared DEGs associated with “defense response” and “immune system response” (Figure 5A,D, Table S8). Shared DEGs associated with “regulation of immune system process” and “response to biotic stimulus” including *Blk, Cfp, Cybb, Fam20a, Il10rb, Il27ra, Lbp, Noct, Rarres2, Rnase4, Saa3*, and *Serping1*, were highly correlated in the liver and spleen (Figure 5B,E, Table S8). Finally, the kidney and spleen indicated high correlation in shared DEGs related to “response to stress” (such as *Acvrl1, Axl, Ccl8, Ccl12, Chaf1a, Col1a1, Cybb, Gzmb, Ier3, Ifi211, Lbp, Pdgfb*, and *Plin2*), as well as “defense response” (Figure 5C,F, Table S7). Furthermore, we assessed expression trends in the three organs and determined that the shared DEGs were enriched in “defense response to other organism” and “innate immune response” (Figure 5D,H, Table S8).

### 2.8 Organ‐Specific Transcriptomic Aging Signatures

To identify age‐associated transcriptional changes uniquely enriched in individual organs, we applied LMM across all tissues, modeling *Age* as a continuous variable and including a random intercept for *Mouse*. An *Age × Tissue* interaction term was used to extract genes with divergent age trajectories between tissues. For each gene, we subsequently ran linear trend tests per organ to identify the direction of expression change (upregulated or downregulated with age) (Figure S4A).

To highlight tissue‐specific aging programs, we extracted genes with significant trends in one organ but not others and performed GO enrichment on these gene sets. This revealed distinct biological aging signals across organs. In the liver, genes upregulated with age were enriched for metabolic processes, including small molecule metabolism, organic acid and lipid metabolism, reflecting heightened metabolic and detoxification demands (Table S8). In contrast, downregulated genes showed strong enrichment for “muscle structure development,” “circulatory system development,” and “vasculature development,” suggesting impaired vascular maintenance and structural remodeling in the aging liver (Table S9). Uniquely age‐upregulated genes in the kidney were enriched for “regulation of cell adhesion,” “positive regulation of cell motility,” and “actin cytoskeleton organization,” suggesting tissue restructuring and potential fibrotic responses with age (Table S9). The downregulated genes in the kidney showed strong signatures of ion and small molecule metabolism and transport processes, including “organic acid metabolic process,” “small molecule metabolic process,” and “organic anion transport,” reflecting a decline in nephron functional capacity and solute handling with age (Table S9). In the spleen, age‐upregulated genes were enriched for mitochondrial and metabolic processes, such as “cellular respiration” and “oxidative phosphorylation,” indicating enhanced mitochondrial adaptation with age (Table S9). In contrast, downregulated genes showed a strong signature of adaptive immune decline, including “lymphocyte activation,” “immune system process,” and “T cell activation,” suggesting immunosenescence primarily driven by reduced lymphocyte function (Table S9). Finally, the unique age‐downregulated genes in the brain showed enrichment in “modulation of chemical synaptic transmission,” “regulation of synaptic plasticity,” and “synaptic signaling,” consistent with age‐associated neural decline and synaptic dysregulation (Table S9).

### 2.9 qPCR‐Based Validation of Selected Differentially Expressed Genes

**FIGURE 6:**
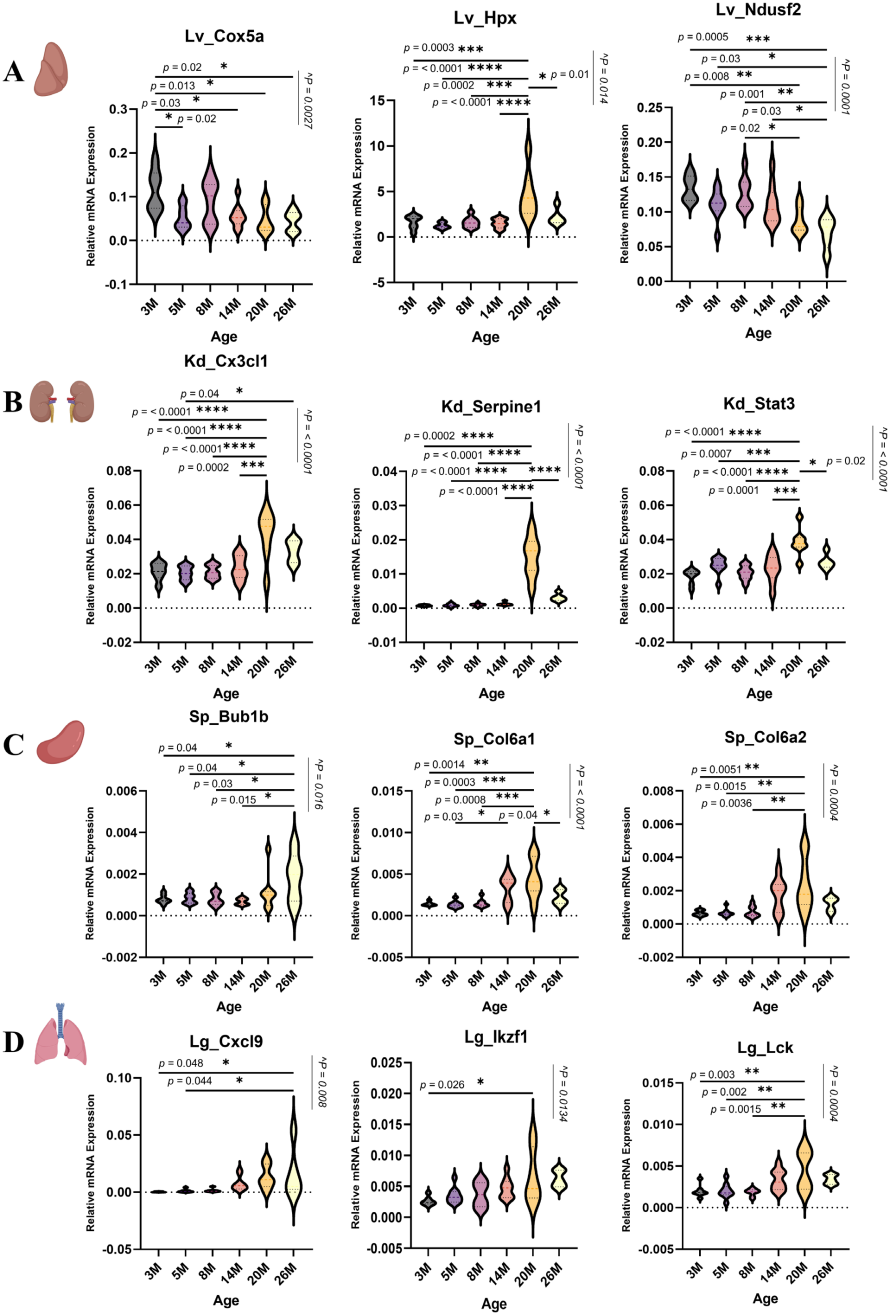
Validation of age‐associated gene expression changes by RT–qPCR. (A–D) Relative mRNA expression levels of selected aging‐associated genes were validated in the (A) liver (Lv), (B) kidney (Kd), (C) spleen (Sp), and (D) lung (Lg) across different age groups. Gene expression was quantified by RT–qPCR, normalized to β‐Actin using the 2^−ΔCT^ method. Violin plots show the distribution of expression levels per group. Violin plot shows upper and lower quartiles (lightly dotted lines) and the median (bold dotted line). Statistical analysis was performed using one‐way ANOVA with age as a between‐subjects factor, followed by Tukey's post hoc test. Significance levels: *p < 0.05; **p < 0.01; ***p < 0.001; ****p < 0.0001. Source data are available online for this figure. ^denotes ANOVA *p* value indicating overall age effect.

Next, we selected a subset of differentially expressed genes from the liver, kidney, spleen, and lung in our bulk RNA‐seq datasets for validation by qPCR (Figure 6A–D). Target genes were selected based on their high expression levels and their enrichment as hub genes among DEGs in any of the four organs. We confirmed the differential gene expression of 28 target candidate genes identified in our bulk RNA‐seq experiments using qPCR (Table S10). The validated DEGs included: 5 genes from the liver (*Cox5a*, *Crp*, *Hpx*, *Ndusf2*, and *Ndusf3*; Figure 6A,S,5), 8 genes from the kidney (*Cdkn1a*, *Csf1, Cx3cl1, Icam1*, *Serpine1*, *Stat3, Tlr4, and Vcam1*; Figure 6B,S,4), 12 genes from the spleen (*Apoe, Bub1b, C1qa, Camp, Ccna2, Col6a1, Col6a2, Csf1r, Fbn1, Mmp2, Nusap1*, and *Vcam1*; Figures 6C,S,4B), and 3 genes from the lung (*Cxcl9, Ikzf1*, *and Lck*; Figure 6D). *Cox5a* and *Ndusf2*, which were also identified among the top age‐associated gene hubs in our liver RNA‐seq dataset, encode genes involved in the mitochondrial electron transport chain (Baertling et al. 2017; Ngu et al. 2012). *Cx3cl1* and *Stat3*, identified among the top age‐associated gene hubs in our kidney RNA‐seq dataset, are implicated in heightened inflammatory responses (Carlsen et al. 2004; Yu et al. 2009). *Bub1b*, *Col6a1*, and *Col6a2* are three of the top gene hubs in our spleen RNA‐seq dataset. *Bub1b* encodes a cell cycle regulator (Davenport et al. 1999), whereas the two collagen genes are involved in ECM remodeling (Vroman et al. 2023). *Cxcl9* and *Lck*, two of the top gene hubs in the lung RNA‐seq dataset, are implicated in chemokine signaling and immune response, respectively (Lin et al. 2004; Ozga et al. 2021). The selected differentially expressed target genes were confirmed by qPCR with validation rates of 50%, 88.80%, 75%, and 60% in the liver, kidney, spleen, and lung, respectively. Validation rates varied across the assessed organs, with the liver and kidney showing the lowest and highest validation rates, respectively. Importantly, the validated target genes showed the same directionality as the RNA‐Seq datasets thereby confirming the reliability of our transcriptomics data.

## 3 Discussion

In this study, we investigated shared and tissue‐specific transcriptomic signatures of aging by performing bulk RNA sequencing on eight major mouse organs‐brain, heart, kidney, liver, lung, skeletal muscle, spleen, and testis. This analysis included 6 age points ranging from 3 to 26 months old mice and therefore encompassing much of the murine lifespan. Unlike previous rodent aging studies, which often focused on single organs, relied on pairwise comparisons, or suffered from small sample sizes, our design incorporated a larger cohort (*N* = 45) and leveraged a likelihood ratio test (LRT) to capture global age‐associated transcriptomic changes (Love et al. 2014). Our analyses revealed that the liver, kidney, and spleen exhibited the most pronounced transcriptomic alterations with aging, while changes were moderate in the heart, lung, and skeletal muscle and minimal in the brain and testis (Figure 1 and Table S1). We further validated a subset of DEGs in the kidney, liver, lung, and spleen using qPCR (Figure 6). Strikingly, the validation rate for the liver was notably lower than the other organs, which is likely to reflect the post‐transcriptional regulatory complexity of this organ. Processes such as alternative polyadenylation and 3′‐UTR remodeling (Jobbins et al. 2022), the dominant influence of the liver‐specific miRNA miR‐122 (Jopling 2012), and age‐related changes in RNA‐binding proteins (Chaturvedi et al. 2015) can decouple isoform‐level signals captured by RNA‐seq from those measured by targeted qPCR assays. The validated differentially expressed genes represented the most enriched gene hubs associated with DEGs across the four organs.

Interestingly, age‐associated differential gene expression patterns (Figure 2A–H) varied across organs, reflecting differences in the onset and extent of transcriptomic alterations. The spleen, lung, skeletal muscle, and testis showed earlier onset of transcriptional alterations starting around 8 months, peaking at 20 months, and declining or stabilizing by 26 months. In contrast, brain, heart, kidney, and liver displayed later‐onset changes beginning around 14 months, also with varied trajectories, either stabilizing or declining afterward. Interestingly, despite the lung, skeletal muscle, and testis's early onset of changes, these organs displayed fewer DEGs, indicating that the timing and magnitude of transcriptional shifts are uncoupled in these organs.

Comparing these transcriptomic patterns to our separately published proteomic data (Scifo et al. 2025) revealed consistent trends in both the timing and extent of molecular changes. Both datasets showed stable expression in the kidney, lung, and spleen until 8 months, while the liver remained stable until 14 months. Subsequently, molecular changes in the kidney, lung, and spleen intensified, peaking at 20 months before declining by 26 months. In contrast, the liver underwent a sharp transition between 14 and 20 months and maintained this altered state thereafter. Notably, the kidney, liver, and spleen consistently exhibited the most pronounced changes across both datasets, while changes in the lung remained moderate. However, a key distinction emerged: transcriptomic changes were generally more extensive than proteomic shifts, highlighting post‐transcriptional regulation as a potential modulator of aging‐related molecular phenotypes. Importantly, beyond similar temporal patterns, we also observed functional convergence between the transcriptomic and proteomic layers. Shared aging signatures included a coordinated upregulation of immune response and complement activation across tissues, particularly in the spleen, alongside a consistent downregulation of translational machinery and proteostasis‐related pathways. Moreover, extracellular matrix remodeling emerged in both datasets, especially in the kidney and spleen, underscoring conserved biological processes driving tissue‐specific aging programs. These overlaps reinforce the robustness of our findings and suggest that key aspects of age‐associated molecular remodeling are captured across omics layers.

Trajectory analysis of age‐related DEGs in various mouse organs, as shown in Figures 3A,B,S,1 and 2A–D, revealed that the onset and progression of transcriptomic changes varied significantly between different organs. A previous multi‐organ transcriptomic study identified enrichment in genes related to increased inflammation, mitochondrial dysfunction, and loss of proteostasis (Schaum et al. 2020) during normal aging. In our study, based on gene ontology analysis of DEGs associated with the most enriched clusters for individual organs, we confirmed that genes related to increased inflammatory response (e.g., *Cxcl13, Stat3* and *Tnf*) (Bradley 2008; Carlsen et al. 2004; Yu et al. 2009) and mitochondrial dysfunction (e.g., *Cox5a, Ndufs5*, and *Uqcrb*) (Baertling et al. 2017; Haut et al. 2003; Salscheider et al. 2022) were also overrepresented in all assessed mouse organs except for the testis. The absence of inflammaging signature in the testis is inconsistent with literature evidence in rodents (Frungieri et al. 2018; Giannessi et al. 2005; Zhang et al. 2020) and may suggest that this organ becomes more susceptible to inflammation much later in life in comparison to the other assessed organs. Moreover, genes involved in ribosome biogenesis and translation (e.g., *Emg1, Rplp0*, and *Rps27*) (Meyer et al. 2011; O'Donohue et al. 2010; Santos and Ballesta 1994), whose dysregulation is implicated in loss of proteostasis (Tye et al. 2019), were prominent in cluster 5 of the spleen.

Our network analysis revealed the most prominent molecular programs associated with age‐related dysfunction in individual organs. In the aging liver, cluster 1 (Figure 3A) highlighted upregulation of Cytochrome P450 enzymes (CYPs), suggesting an adaptive response to increased metabolic demands and detoxification pressures, possibly serving as a compensatory mechanism against oxidative stress (Rendic 2021). In addition, genes encoding apolipoproteins, acute‐phase proteins, and glutathione‐S‐transferases were enriched in cluster 1, further indicating the complex dynamics of metabolic adaptation, oxidative stress management, and inflammatory responses during aging. Surprisingly, cluster 2 (Figure 3A) of the liver uncovered an overrepresentation of genes typically associated with neuronal signaling, including AMPA, GABA, NMDAR, and adrenergic receptors‐rarely studied in hepatic aging. While these genes are well known for their roles in the nervous system, there is also evidence that they function in non‐neuronal tissues. For instance, hepatocyte‐driven glutamate release can activate mGluR5 on hepatic stellate cells, promoting fibrogenesis (Choi et al. 2019). GABAα receptors, conversely, appear to be protective‐mitigating hepatocyte apoptosis and inflammation (Wang et al. 2017). Similarly, NMDAR subunits, scarcely expressed in healthy liver, were previously shown to be upregulated in fibrosis and hepatocellular carcinoma models (Stepanov et al. 2022). The upregulation of α1‐ and α2B‐adrenergic receptors aligns with stellate cell activation and fibrotic remodeling (Schwinghammer et al. 2020). Together, these findings suggest a pathological shift where aberrant glutamatergic and dopaminergic signaling contribute to fibrosis and malignancy, while GABAergic pathways act as a protective counterbalance. The liver's cluster 3 (Figure 3A) reflected declining mitochondrial integrity, with downregulation of mitochondrial ribosomal genes and components of the electron transport chain and TCA cycle, pointing to impaired mitochondrial protein synthesis and bioenergetic stress (Cheong et al. 2020). Concurrent suppression of *Prkar1a*, a key PKA signaling regulator (Robinson‐White et al. 2006), and *Cs* (citrate synthase) underscores broader disruptions in metabolic control. Reduced expression of antioxidant defenses (*Prdx3* and *Sod2*) (Al‐Serri et al. 2012; Geng et al. 2021) and mitochondrial biogenesis regulators (*Ppargc1a* and *Tfam*) (Collier et al. 2024) further signals compromised mitochondrial renewal and heightened oxidative vulnerability, hallmarks of liver aging.

In the kidney, the aging signature similarly pivoted around oxidative stress, inflammation, and cellular senescence. Cluster 2 (Figure 3B) captured the metabolic decline, with loss of Cystathionine beta‐synthase (CBS), a critical enzyme in sulfur amino acid metabolism and linked to oxidative stress and chronic kidney disease (CKD) (Hamidizad et al. 2023), and *Sirt3*, a regulator of mitochondrial ROS through Superoxide dismutase (SOD2) (Chen et al. 2011). Peroxisomal acyl‐coenzyme A oxidase 1 (ACOX1), a key enzyme in peroxisomal fatty acid oxidation, was shown to be downregulated in renal biopsies from acute kidney injury and CKD patients (Chen et al. 2023), while *Ppargc1a* suppression reflects failing mitochondrial biogenesis and energy metabolism, as observed in diabetic kidney disease (She et al. 2022). *Il6, Stat3* and *Tnf*, which were enriched in cluster 3 (Figure 3B) of the aging kidney, have been linked to renal inflammation and fibrosis, contributing to CKD progression through excessive cytokine signaling and extracellular matrix accumulation (Kandemir et al. 2022; Zheng et al. 2019). Notably, the parallel rise in *Il10ra* suggests an attempted, yet likely insufficient, anti‐inflammatory counterbalance (Saraiva and O'Garra 2010). The concurrent increase in senescence markers (*Cdkn1a* and *Cdkn2a*) is in line with an increasing senescent cell load in the aging kidney (Khosla et al. 2020). The data suggest a critical tipping point between 14 and 20 months, where compensatory mechanisms fail and may lead to senescence associated with pathology and deterioration. This complex interplay of stress responses, inflammation, and aging underscores the kidney's vulnerability and positions it as a central organ for age‐related disease progression.

In the spleen, network analysis revealed progressive fibrotic remodeling and immune alterations driving aging‐related dysfunction. Enrichment of collagen genes and ECM components (Cluster 1) implies structural adaptation or maladaptation (Ricard‐Blum et al. 2018), while the decline in adaptive immune genes (Cluster 2) hints at functional impairment of adaptive immunity associated with aging (Figure S1) (Zhou et al. 2021). Cell cycle regulators represented by genes such as *Aurkb, Bub1b, Ccnb1, Cdc20, Chek1*, and *Plk1*, enriched in cluster 3 (Figure S1) of the spleen, are critical for mitotic progression and checkpoint control (Abe et al. 2018; Davenport et al. 1999; Hein et al. 2021; Schmucker and Sumara 2014; Shaalan et al. 2021; Strauss et al. 2018). The concurrent upregulation of *Birc5* (Survivin) and *Cdkn1a* among the enriched genes in this cluster further indicates a potential role of the former in regulating senescence in the aging spleen (Unruhe et al. 2016). The prominence of *Pdgfra* and *Pdgfrb* in cluster 4 (Figure S1) of the spleen underscores the activation of platelet‐derived growth‐factor (PDGF) signaling, a major driver of fibroblast proliferation and ECM deposition, aligning with findings in murine myelofibrosis models (Herrera et al. 2018). Likewise, the TGF‐β pathway, represented by *Tgfb2* and *Tgfbr1*, emerges as a key pro‐fibrotic regulator in the aging spleen. Structural ECM components (e.g., *Col1a1* and *Col3a1*) and matrix regulators (e.g., *Adamts5* and *Serpine1*) in the cluster are consistent with extensive matrix remodeling and fibrotic maladaptation in the aging spleen (Barallobre‐Barreiro et al. 2021). Finally, Cluster 5 (Figure S1) revealed downregulation of genes (e.g., *Abce1, Efl1, Ngdn*, and *Rplp1*) implicated in the ribosomal and translation machinery, suggesting that impaired protein synthesis, ribosome maturation defects, and declining translational capacity may represent key features of the aging spleen (Campos et al. 2020; Jung et al. 2006; Nurenberg‐Goloub et al. 2020; O'Donohue et al. 2010).

Prominent clusters in the lungs were enriched in immune response genes, inflammatory mediators and chemokines (cluster 1), as well as metabolism and detoxification related genes (cluster 2) (Figure S2A). Progressive activation of myeloid cells, characterized by increased expression of immune response genes (*Cybb, Hck*, and *Itgam*) (Bai et al. 2022), inflammatory mediators (e.g., *Alox5* and *Nlrp12*) (Poirier et al. 2020; Udden et al. 2019) and elevated chemokine signaling genes (*Ccr5, Cx3cr1*, and *Cxcl9*) (Ozga et al. 2021), indicates sustained inflammaging in the lung. In contrast, adaptive cell mediated immune response genes show a dual pattern of activation (*Cd3g, Il7, Itk*, and *Lck*) (Lin et al. 2004; Panapasa et al. 2015) and regulation (*Cd22, Ctla4* and *Ikzf1*) (Ichiyama et al. 2024; Kawasaki et al. 2011; Vignali et al. 2008), suggesting a shift toward functional dysregulation with age. Interestingly, metabolic and detoxification genes (*Cyp1a2, Hao1*, and *Hao2*) remained stable until late life, declining at 26 months, hinting at an initial metabolic robustness that deteriorates with advanced age (Isobe et al. 2018; Kalidasan et al. 2024; Stading et al. 2021).

In skeletal muscle, aging was associated with ECM remodeling, metabolic dysregulation, and immune activation. Cluster 1 (Figure S2B) highlighted upregulation of collagen genes (*Col1a1* and *Col6a1*) (Vroman et al. 2023) and metabolic regulators (*Amd1, Amd2, Odc1, Pdk4, Smox*, and *Ucp3*) (Furbish et al. 2022; Lee and MacLean 2011; MacLellan et al. 2005; Sugden and Holness 2003), indicating structural compromise and altered energy homeostasis. The presence of inflammatory and immune‐related genes (e.g., *Ccl8, Il1b*, and *Slfn4*) in cluster 2 (Figure S2B) may indicate heightened immune activation in aging muscle (Ding et al. 2016; Laurance et al. 2010). In addition, the presence of stress response and metabolic regulators (*Gpx3, Klf5*, and *Osmr*) (Chung et al. 2009; Domaniku‐Waraich et al. 2024; Hayashi et al. 2016) which play roles in tissue remodeling together with ECM and muscle homeostasis genes (*Ctss* and *Lgmn*) (Osoble et al. 2025; Wan et al. 2023), hint at remodeling processes essential for muscle function and repair. Yet, the upregulation of *Cdkn1a* suggests that accumulating senescent cells contribute to impaired regeneration and muscle wasting.

In the heart, transcriptomic profiling revealed a convergence of innate immune activation, chronic inflammation, and cellular senescence as prominent aging features. Clusters 1 and 3 (Figure S2C) were enriched in genes linked to macrophage and dendritic cell activation (*Aim2, Ccr1, Ccr2*, and *Tlr4*), reflecting heightened innate immune engagement (Bajpai et al. 2019; Medina‐Ruiz et al. 2024; Wang et al. 2020). This was accompanied by upregulation of proinflammatory chemokines (*Cxcl2, Cxcl5*, and *Cxcl13*), sustaining a chronic inflammatory state that is commonly associated with cardiac damage (Chen et al. 2022). The co‐expression of adaptive and innate immune modulators (*Fcgr2b, H2‐Q7, Nlrc5*, and *Ptprc*) alongside senescence markers (*Cdkn1a* and *Serpina3n*) suggests a maladaptive remodeling process, where persistent immune activation transitions toward immune exhaustion and tissue deterioration (Al Barashdi et al. 2021; Ba et al. 2022; Ludigs et al. 2015; Melo‐Lima et al. 2014; Morris et al. 2020; Poh et al. 2012). Interestingly, downregulation of oxygen transport (*Hbb‐bs*) and vascular function (*Alas2*) genes suggests impaired oxygen delivery and endothelial homeostasis shift may represent a compensatory adaptation aimed at reducing excess heme accumulation and limiting ischemic injury in aging cardiomyocytes (Sawicki et al. 2015; Zhang et al. 2023). Together, these findings depict a scenario where sustained innate immune activation, unresolved inflammation, immune exhaustion, and senescence promote cardiac aging, contributing to structural remodeling and functional decline.

Finally, the aging brain displayed transcriptional signs of chronic neuro‐inflammation and microglial activation. Cluster 2 (Figure S2D) showed upregulation of *Cd74, Clec7a, Cst7*, and *Cybb*‐genes implicated in reactive microglia and neurodegenerative processes (Potru and Spittau 2023). *Cybb*, encoding the NADPH oxidase component gp91‐phox, likely exacerbates oxidative stress, driving neuronal damage (Valencia et al. 2013). *Cst7*, a lysosomal protease inhibitor, has been shown to modulate microglial inflammatory responses in tauopathy models, while *Clec7a* (Dectin‐1) mediates amyloid‐beta phagocytosis (Rasmussen et al. 2022). Cd74, which interacts with macrophage migration inhibitory factor (MIF), is linked to Alzheimer's pathology through amplification of inflammatory pathways (Bryan et al. 2008). Overall, these findings highlight a recurring theme of immune dysregulation, fibrotic remodeling, metabolic stress, and senescence as key hallmarks shaping the aging trajectories of multiple organs‐each with unique timing and intensity reflective of their biological roles and vulnerabilities.

We identified genes encoding vascular growth factors (e.g., *Edn1, Flt1, Kdr*, and *Pdgfb*) (Eilken et al. 2017; Waltenberger et al. 1999), innate immune triggers (*Ccl5, Ccl8, Ccl12, Cxcl1, Cxcl13, Nfkb1*, and *Tlr4*) (Richmond 2002; Wang et al. 2020), complement factors (*C3* and *C4b*) (Noris and Remuzzi 2013) as well as B‐ and T‐Cell markers (*Birc3, Cd22, Cr2, Fcgr4*, and *Irf7*) (Boldison et al. 2023; Kawasaki et al. 2011), among shared age‐related DEGs between the kidney, liver, and spleen, all of which point to chronic low‐grade inflammation (inflammaging), vascular, immune, and tissue remodeling as critical aspects of aging in the three organs. In addition, oxidative stress markers (*Cybb, Nox4*, and *Sod2*) (Isiaku et al. 2024; Zhang et al. 2022), collagens (e.g., *Col1a1* and *Col6a1*) (Vroman et al. 2023), fibrosis regulators (e.g., *Fbn1* and *Thbs1*) (Tanaka et al. 2021; Wu et al. 2024), and matrix metalloproteinases (*Mmp2* and *Mmp9*) (Cancemi et al. 2020) constituted part of their shared transcriptomic landscape. Yet, despite these shared molecular themes, the directionality of gene expression was often organ‐specific. Notably, fibrotic, vascular, and immune genes were downregulated in the liver at 20 months, contrasting with their upregulation in the kidney and spleen. This divergence highlights that aging is not a uniform process but rather governed by tissue‐specific regulatory programs. Whereas the kidney's trajectory reflects progressive fibrosis and structural stiffening, the liver may shift toward ECM degradation and heightened inflammatory reactivity, emphasizing the need for organ‐tailored aging interventions.

In contrast to existing multi‐organ aging datasets, which are often limited by small sample sizes and analyses based on pairwise comparisons between older mice and a young adult reference (Schaum et al. 2020; Takasugi et al. 2024), our study provides a complementary resource for in‐depth assessment of age‐dependent changes. Our work indicates that normal aging progresses in gradual and non‐linear ways across different tissues. Although there is an overlap of age‐related genes across organs, we confirm that the age of onset and magnitude of transcriptomic changes vary between the different tissues covered in our analysis. A limitation of the study is that our investigations were performed in one sex and one genetic background (male C57BL/6J mice). It would be desirable to extend our findings to female mice, as well as additional mouse strains.

In conclusion, we identified dominant age‐associated dysregulated transcriptomic signatures across multiple organs, including progressive metabolic decline and fibrosis in the kidney; alterations in mitochondrial metabolic pathways and distinct late‐life downregulation of ECM and fibrotic genes in the liver; changes in inflammatory and detoxification processes in the lung; and dysregulation of angiogenesis, vascular remodeling, ribosome biogenesis, and translation in the spleen. Moreover, we also identified increased inflammatory response, mitochondrial dysfunction and cellular senescence as the most prominent aging features seen across most organs assessed. In addition, evidence for loss of proteostasis was found in the aging spleen. These findings highlight both unique and shared transcriptomic signatures associated with aging across individual and multiple organs, respectively, which could inform the development of therapeutics aimed at improving health in the context of aging. Our findings emphasize the need for organ‐targeted approaches in aging research and therapeutic development, as molecular aging is not uniform but instead reflects distinct vulnerabilities and compensatory mechanisms within each tissue.

## 4 Materials and Methods

### 4.1 Animal Housing and Husbandry Conditions

Our study involved male C57BL/6J mice (stock no. 632, Jackson Laboratory), spanning six age groups: 3, 5, 8, 14, 20, and 26 months. The number of mice used per age group ranged from 7 to 9, except for the 26 months group with 5 mice. All animals were obtained in a single cohort from the Jackson Laboratory. Mice were housed in groups of 2–5 age‐matched individuals per cage in individually ventilated cages (IVCs), maintained under specific pathogen‐free conditions in accordance with FELASA guidelines. Husbandry conditions were standardized at 22°C, 55% humidity, and a 12‐h light/dark cycle, with unrestricted access to food and water. All procedures adhered to local and federal animal welfare regulations. Animals for which predefined humane endpoints, for example, with ulcerating tumors, bleeding from orifice or persistent rectal prolapse were identified, were euthanized. We employed the G*Power software (v3.1.9.2) to estimate sample sizes of the mouse cohorts as required for ethical approvals of animal experiments.

### 4.2 RNA Extraction, RNA‐Seq and Data Processing

Tissues were collected over the course of two consecutive days. The number of animals processed each day was evenly distributed across the different age groups. Animals were given a minimum of 30 min to acclimate in the dissection room before euthanasia and tissue collection. Mouse organs were pulverized in liquid nitrogen to obtain homogenous tissue powder. Total RNA isolation from mouse tissues was performed with TRI‐Reagent (Merck, Darmstadt, Germany). Briefly, 1 mL TRI‐Reagent was added to an aliquot of frozen tissue powder followed by solubilization via ten passages through a 24‐gauge needle. Further processing steps were performed according to the manufacturer's recommendations. Total RNA was purified with the Monarch RNA Cleanup kit (New England Biolabs, Ipswich, MA, US).

A previously described protocol (Hou et al. 2015) was used for mRNA isolation and cDNA library preparation with a few modifications. Briefly, mRNA was isolated from purified 1 μg total RNA using oligo‐dT beads (New England Biolabs, Ipswich, MA, US) and fragmented in reverse transcription buffer by incubating at 85°C for 7 min, before cooling on ice. SmartScribe reverse transcriptase (Taraka Bio, Kusatsu, Japan) with a random hexamer oligo (HZG883: CCTTGGCACCCGAGAATTCCANNNNNN) was used for cDNA synthesis. Samples were then treated with RNase A and RNase H to remove RNA, followed by purification of cDNA on Agencourt AMPure XP beads (Beckman Coulter, Brea, CA, US). The single stranded cDNA was ligated with a partial Illumina 5′ adaptor (HZG885:/5phos/AGATCGGAAGAGCGTCGTGTAGGGAAAGAGTGTddC) using T4 RNA ligase 1 (New England Biolabs, Ipswich, MA, US) and incubated overnight at 22°C. Ligated cDNA was purified on AMPure XP beads and amplified by 20 cycles of PCR using FailSafe PCR enzyme (Epicenter Technologies, Thane, India) and oligos that contain full Illumina adaptors (LC056: AATGATACGGCGACCACCGAGATCTACACTCTTTCCCTACACGACGCTCTTCCGATCT and unique index primers: CAAGCAGAAGACGGCATACGAGATnnnnnnnnnnGTGACTGGAGTTCCTTGGCACCCGAGAATTCCA, where nnnnnnnnnn indicates index nucleotides) for each sample. The resulting cDNA libraries were purified on AMPure XP beads, size selected using SPRIselect beads (Beckman Coulter, Brea, CA, US), and quantified by Qubit dsDNA HS Assay Kit (Thermo Fisher Scientific, Waltham, MA, US) prior to pooling. The pooled library was run on an Agilent High Sensitivity DNA chip (Agilent Technologies, Santa Clara, CA, US) with an Agilent 2100 Bioanalyzer instrument (Agilent Technologies, Santa Clara, CA, US) to check the quality and average fragment size.

Pooled indexed cDNA libraries were sequenced on an Illumina NovaSeq 6000 system (Illumina, San Diego, CA, US) with a single 111 bp read and 10 bp index read. Demultiplexing and data transformation to generate fastq files was done using bcl2fastq2 (v2.20). Sequencing reads were trimmed using CutAdapt (https://usegalaxy.org/) to remove adapter sequences. Trimmed reads were mapped to the mouse transcriptome (GRCm38, mm10) using HISAT2 (v2.1.0) in Galaxy (https://usegalaxy.org/) with forward strand information and default settings. Bam files were indexed using Samtools and count matrices generated by Genomic Alignments in R. Gene count matrices were generated using annotation information from a Mus_musculus.GRCm38.102.chr.gtf file imported with the rtracklayer (Lawrence et al. 2009) package into R. All downstream analyses were performed using R (Version 3.5.1, https://cran.r‐project.org/). Library normalization and expression differences between samples were quantified using the DESeq2 package (Love et al. 2014). A false discovery rate (FDR) < 0.05 was used as a cutoff in differential expression analyses. We also re‐analyzed 2 previously published datasets from the same mice for the spleen and brain (GSE168068), for comparison of age‐related transcriptomic changes across organs. Primer sequences used for the bulk RNA sequencing experiments are listed in Table S11.

### 4.3 UpSet Plot Analysis

Differentially expressed genes identified in the 8 studied mouse organs (brain, heart, lung, liver, kidney, spleen, skeletal muscle, and testis) with an overall *p*‐value ≤ 0.05 across 6 age groups (3, 5, 8, 14, 20, and 26 months) were used as input data for the Upset plot. DESeq2 package was employed for library normalization and differential expression analysis across samples was based on the likelihood ratio test (LRT) to compute overall *p*‐values across all age groups without specifying a reference. Graphical representation of the Upset plot was performed using the UpSetR (v1.4.0) package in R v4.3.1. Shared and unique organ‐specific genes are shown as connected or single dots, respectively. DEGs which are unique or shared across one or more organs are depicted with black dots (Figure 1D).

### 4.4 Trajectory Analysis

We estimated gene expression trajectories during normal aging by using log‐transformed normalized counts from our RNA‐seq datasets. The normalized counts of individual genes from each mouse organ at the various time points were first subjected to z‐score normalization using the “scale” function in R and the average values of z‐scored normalized counts subsequently used as input data for the time series analysis. Gene expression dynamics across the organs were categorized into 2–5 clusters associated with normal aging on the basis of their partition coefficient (PC) and partition entropy (PE), using the fuzzy c‐means algorithm of the Mfuzz package (v2.62.0) in R v4.3.1, with the optimal fuzzifier determined by the “mestimate” function (Futschik and Carlisle 2005; Kumar and Futschik 2007). To identify unique and shared gene expression trajectories associated with normal aging across the 7 assessed mouse organs, we plotted the most enriched clusters from individual organs on a single graph. This allowed us to visualize and categorize clusters with similar or unique gene expression dynamics. We then performed GO analysis in STRING (https://string‐db.org/) to identify biological processes and functions associated with each cluster (Table S4). The testis with only 29 DEGs was not subjected to trajectory analysis.

### 4.5 Construction of Gene Network Hubs

Age‐related DEGs for the liver, kidney, spleen, lung, and skeletal muscle were input into Cytoscape (v.3.10.0, https://cytoscape.org/) to determine the top 500 (liver), 100 (kidney and spleen‐except for cluster 5 (50)), or 30 (lung, cluster 1), 20 (lung, cluster 2), and skeletal muscle (all genes in clusters 1 and 2) gene hubs per cluster based on maximal clique centrality (MCC). Networks for the top gene hubs of the liver, kidney, spleen, lung, and skeletal muscle were visualized in STRING. STRING interaction networks for the top hub genes were identified using CytoHubba, a plug‐in of Cytoscape. Hub genes were identified according to the number of associations with other genes in the network.

### 4.6 Senescence Pathway Analysis Across Organs

Bulk RNA‐seq expression matrices were log2 (x + 1) transformed per organ. Ages were encoded as numeric months. We computed GSVA scores (Gaussian kernel) (Hänzelmann et al. 2013) for the SenMyo gene set per sample and organ (GSVA v2.0.7). For each organ, we fit

$$\text{SenMyo}\sim {\mathrm{Age}}_{c}\times \text{Organ}+\left(1|\text{MouseID}\right)$$

with a random intercept for Mouse. We tested non‐linearity with GAMs (mgcv (v. 1.9.3); REML; (k = 5)) and report *p* values for the smooth term, effective degrees of freedom, the Spearman rank correlation (SenMyo vs. Age) to summarize the directionality, and Delta 26 M‐3 M as an interpretable effect size. Within each organ we computed Spearman *ρ* between SenMyo GSVA and *Cdkn1a*/*Cdkn2a* expression across samples; *p* values were FDR‐adjusted. For SenMyo per organ representative genes we computed: 1. Age association: Spearman *ρ* (gene vs. Age) with Benjamini–Hochberg FDR. 2. Gene–module coherence: Pearson R (gene vs. SenMyo GSVA). A gene qualified as a driver if FDR_age < 0.05 and sign (R) matched its age direction (Up: *R* > 0; Down: *R* < 0). For each organ, we correlated every SenMyo gene with *Cdkn1a* or *Cdkn2a* (Pearson R), computed FDR across genes, and plotted R vs. –log10(FDR), labeling the top hits by |R|.\n

### 4.7 Analysis of Tissue Specific Age‐Related Gene Expression Dynamics by Linear Mixed Modeling

To identify genes exhibiting tissue‐specific age‐related expression dynamics, we applied LMM across transcriptomic data from eight mouse organs. Expression data were transformed into long format, capturing sample‐wise gene expression alongside metadata for tissue, age, and mouse ID. The model was defined as:

$$\text{Expression}\sim {\mathrm{Age}}^{*}\;\text{Tissue}+\left(1\;|\;\text{Mouse}\right)$$

Here, Age was modeled as a continuous variable to capture age‐associated trends, and Tissue was a categorical variable to account for baseline expression differences across organs. The Age × Tissue interaction term was used to detect tissue‐specific age effects. A random intercept for Mouse was included to account for within‐subject correlation arising from multi‐organ sampling of the same animals. For each gene, we compared the full model (with the Age × Tissue interaction) to a reduced model (excluding the interaction term) using a likelihood ratio test (LRT). The resulting *p* values were adjusted for multiple testing using the Benjamini–Hochberg procedure, and genes with adjusted *p* values < 0.05 were considered to show significant tissue‐specific age‐related expression changes.

For genes exhibiting significant Age × Tissue interaction effects in the LMM, we conducted a follow‐up organ‐wise linear trend analysis to determine the directionality of age‐associated expression changes within each tissue. Specifically, for each gene‐tissue pair, we fitted a simple linear regression model:

Expression~Age

Age was treated as a continuous variable, and the significance of the slope was assessed using the *p*‐value associated with the age coefficient. These *p* values were adjusted for multiple testing using the Benjamini–Hochberg method. Genes with a significant slope (adjusted *p* < 0.05) were labeled as Upregulated (positive slope) or Downregulated (negative slope) within that tissue; otherwise, the gene was marked as No Change. This approach allowed us to construct a directionality matrix, capturing tissue‐specific transcriptional aging trends across the gene set identified by the LMM.

### 4.8 Real‐Time Quantitative PCR

Total RNA was reverse transcribed into cDNA using the iScript cDNA Synthesis Kit (Bio‐Rad, Hercules, CA, USA). Quantitative real‐time PCR was carried out with PowerUP SYBR Green Master Mix (Thermo Fisher Scientific, Waltham, MA, USA) on a StepOnePlus Real‐Time PCR System (Thermo Fisher Scientific). Gene expression levels were normalized to β‐actin (Actb) using the comparative Ct method. Primer sequences are listed in Table S10.

### 4.9 Quantification and Statistical Analysis

Statistical analysis details, including the tests and N number used, are provided in the figure legends.

## Author Contributions

Dan Ehninger conceived the research project and provided supervision; Dan Ehninger, Enzo Scifo, and Kan Xie planned the research project; Sarah Morsy, Enzo Scifo, and Kan Xie performed experiments; Sarah Morsy, Enzo Scifo, and Kan Xie analyzed data; Dan Ehninger, Sarah Morsy, Enzo Scifo, and Daniele Bano contributed interpretation and discussion; Kristina Schaaf and Kan Xie provided technical and scientific support; Sarah Morsy, Enzo Scifo, and Dan Ehninger drafted the manuscript; Daniele Bano and Dan Ehninger provided resources. All authors agreed on the final version of the paper.

## Funding

This work was supported by HORIZON EUROPE Marie Sklodowska‐Curie Actions (101072759).

## Disclosure

Data and Code Availability: Raw data are available on GEO (GSE291420). Analysis code is available at: https://github.com/ehningerd/Morsy‐Scifo_et_al‐mouse_aging_transcriptome.

## Conflicts of Interest

The authors declare no conflicts of interest.

## Supporting information


**Figure S1:** Temporal gene expression clusters and hub gene networks in the spleen. Mfuzz clustering of z‐scored expression profiles of age‐associated differentially expressed genes (DEGs) in the spleen, illustrating distinct temporal trajectories. Each panel depicts the average cluster trend (bold line) alongside individual gene profiles. Adjacent to each trajectory plot, protein–protein interaction networks of the highest‐ranked hub genes are shown. Hub genes were defined by maximal clique centrality (MCC) scores using CytoHubba and visualized in STRING based on STRING interaction data. Nodes represent genes and edges denote functional associations, with densely connected nodes (“hubs”) indicating potential key regulators. Top 100 genes per cluster are shown except for cluster 5 where we show the top 50 gene hubs.

**Figure S2:** Age‐associated gene expression trajectories and Organ‐Specific Transcriptomic Directionality (A–D) Mfuzz clustering of z‐scored expression profiles of age‐associated differentially expressed genes (DEGs) in lung (A), skeletal muscle (B), heart (C), and brain (D), revealing organ‐specific temporal trajectories during aging. Each cluster panel shows the mean expression trend of individual gene profiles. Protein–protein interaction networks of top hub genes are displayed adjacent to cluster plots, except for heart and brain, where the low number of DEGs precluded network construction. Hub genes were ranked by maximal clique centrality (MCC) using CytoHubba and visualized in STRING based on STRING interaction data. Nodes represent genes and edges indicate functional associations, with highly connected “hubs” potentially serving as key regulatory points. For network visualization, the following hub gene sets were used: lung ‐ top 30 genes for cluster 1 and top 20 for cluster 3; skeletal muscle ‐ all genes in clusters 1 and 2. (E) Tissue‐specific directionality of age‐related transcriptional changes derived from linear mixed‐effects modeling (LMM). Heatmap showing genes with significant Age‐by‐Tissue interaction effects (LMM, adjusted *p* < 0.05), followed by linear trend tests within each organ to determine directionality. Rows represent individual genes and columns represent organs. Colors indicate age‐associated expression changes: red = upregulated, blue = downregulated, and white = not significantly changed within each tissue. The matrix highlights both consistent and divergent aging trajectories across organs.

**Figure S3:** Organ‐specific dynamics and drivers of the senescence/myofibroblast program (SenMyo) (A) LMM age slopes (per month). Estimated age effects of SenMyo activity (GSVA score) from a linear mixed model: SenMyo ~ Age × Organ + (1|MouseID). Bars show slope (GSVA units/month) ±95% CI from ‘emtrends’. Kidney and spleen show the steepest increases; liver is ~0/slightly negative. (B) SenMyo activity across age. Mean (±SE) SenMyo GSVA scores per age group and organ, computed with GSVA (Gaussian kernel) on the full transcriptome within each organ. Trajectories highlight early/strong rise in kidney, later/moderate rises in spleen/heart/brain, late shift in skeletal muscle, and flat profiles in lung and liver. (C) GAM summary per organ. For each organ, a GAM was fit to SenMyo vs. age (GAM: SenMyo ~ s(Age), REML). Point position = Spearman *ρ* (SenMyo vs. age); point size = −log10(*p* for the smooth term; capped for display); color encodes the sign of *ρ*; labels show Δ = mean change (26 M–3 M). (D) Heatmap of the top 20 age‐upregulated genes per organ that are coherent with the module. Each gene met (i) significant gene–age association (Spearman FDR < 0.05 within organ) and (ii) positive coherence with SenMyo (Pearson R between gene expression and SenMyo across samples); ranking = |R| × |*ρ*_age| × −log10(FDR_age). (E) Heatmap of Spearman correlations between SenMyo GSVA and Cdkn1a or Cdkn2a (p16) per organ. (F) Per‐gene Pearson correlations with markers. For each organ and marker (columns within facets), scatter of gene–marker Pearson correlations (R, x‐axis) vs. –log10(FDR) for the gene's age association (y‐axis). Points are colored by correlation sign (red = positive, blue = negative); select genes are labeled.

**Figure S4:** Validation of age‐associated gene expression changes in the spleen. RT–qPCR analysis of selected aging‐associated genes in the spleen across different age groups. Gene expression levels were normalized to β‐actin using the 2^−ΔCT^ method. Violin plots display the distribution of relative mRNA expression per group, with mean ± SEM indicated. Violin plot shows upper and lower quartiles (lightly dotted lines) and the median (bold dotted line). Statistical analysis was performed using one‐way ANOVA with age as a between‐subjects factor, followed by Tukey's post hoc test. Significance levels: *p < 0.05; **p < 0.01; ***p < 0.001; ****p < 0.0001. ^denotes ANOVA *p* value indicating overall age effect.

**Figure S5:** Validation of age‐associated gene expression changes in the kidney and liver. RT–qPCR analysis of selected aging‐associated genes in the kidney and liver across different age groups. Gene expression levels were normalized to β‐actin using the 2^−ΔCT^ method. Violin plots display the distribution of relative mRNA expression per group, with mean ± SEM indicated. Violin plot shows upper and lower quartiles (lightly dotted lines) and the median (bold dotted line). Statistical analysis was performed using one‐way ANOVA with age as a between‐subjects factor, followed by Tukey's post hoc test. Significance levels: *p < 0.05; **p < 0.01; ***p < 0.001; ****p < 0.0001. ^denotes ANOVA *p* value indicating overall age effect.

**Table S1:** Total identified genes across mouse organs during normal aging. The DESeq2 package was employed for library normalization and differential expression analysis across samples. Log‐transformed normalized abundances of the 8 assessed mouse organs including the brain, heart, lung, liver, kidney, spleen, skeletal muscle, and testis, are shown. Organs were harvested from male C57BL/6J mice at 3, 5, 8, 14, 20 and 26 months. Abbreviations: Lv‐ liver, Kd‐ kidney, Sp‐ spleen, Lg‐ lung, SkM‐ skeletal muscle, Ht‐ heart, Br‐ brain and Ts‐ testis; 3, 5, 8, 14, 20 & 26 M‐ denotes age in months.

**Table S2:** Summary of differentially expressed genes (DEGs) across mouse organs and age groups. The DESeq2 package was employed for library normalization and differential expression analysis across samples using the likelihood ratio test (LRT) to compute overall *p* values across all age groups without specifying a reference. Genes with an adjusted *p* value ≤ 0.05 were determined to be significantly differentially expressed. The 8 assessed mouse organs include the liver, kidney, spleen, lung, skeletal muscle, heart, brain, and testis. Organs were harvested from male C57BL/6J mice at 3, 5, 8, 14, 20 and 26 months.

**Table S3:** Principal component analysis of age‐related differentially expressed genes for the 8 assessed mouse organs. The principle components (PC), standard deviation, proportion of variance and cumulative proportion are indicated for the mouse organs liver, kidney, spleen, lung, skeletal muscle, heart, brain, and testis.

**Table S4:** Functional annotation of organ clusters for the liver, kidney, spleen, lung, skeletal muscle, heart and brain. Gene ontology enrichment analysis of the most enriched clusters for the 7 assessed mouse organs was done in STRING. GO analysis was done for 5 clusters (spleen), 3 clusters (liver), 2 clusters (heart, kidney, lung and skeletal muscle) and 1 cluster (brain).

**Table S5:** Organ‐resolved senescence module analysis. Mouse genes comprising the SenMyo senescence module, as defined in Saul et al., Nat Commun (Saul et al. 2022), which we used as the basis for downstream scoring are indicated in (A). GSVA‐derived SenMyo module activity scores for each sample and organ across age are shown in (B). Linear mixed‐effects model (LMM) testing age‐dependent changes in SenMyo activity, including fixed effects of organ, an Age × Organ interaction, and mouse‐specific random intercepts are summarized in (C). Generalized additive model (GAM) fits for each organ, capturing non‐linear SenMyo trajectories with age are indicated in (D). The full ranked matrix of genes contributing to SenMyo progression in each organ, and (ii) the top 20 age‐upregulated SenMyo genes per organ, based on joint association with age and concordance with SenMyo activity are shown in (E). The correlation between SenMyo GSVA scores and canonical senescence markers *Cdkn1a* and *Cdkn2a* in each organ is indicated in (F). Gene‐level Pearson correlations between each individual SenMyo gene and Cdkn1a or Cdkn2a, by organ, highlighting organ‐specific coupling between canonical checkpoints and module components are reported in (G).

**Table S6:** Gene hubs and functional annotation for DEGs in the aging liver, kidney, spleen and lung. Gene ontology enrichment analysis of the most enriched gene hubs for the 4 assessed mouse organs was done in STRING.

**Table S7:** Gene hubs and functional annotation of shared DEGs in the aging kidney, liver, and spleen. Gene ontology enrichment analysis of the most enriched gene hubs for the 3 assessed mouse organs was done in STRING.

**Table S8:** Correlation analysis and functional annotation of shared gene hubs kidney‐liver, kidney‐spleen, liver‐spleen and liver, kidney and spleen. Shared DEGs between organ pairs were assessed for correlation of gene changes using Spearman's rank correlation coefficient. Gene ontology enrichment analysis of the most enriched gene hubs for the 3 mouse organ pairs was done in STRING. Abbreviations: Kd‐ kidney, Lv‐ liver, and Sp‐ spleen.

**Table S9:** Linear mixed‐effects modeling and tissue‐specific trend analysis across eight murine organs. Significant genes determined by the linear mixed‐effects model (LMM), matrix summarizing the directionality of age‐related gene expression and Gene ontology (GO) enrichment analysis, are indicated. Abbreviations: Br‐ brain, Kd‐ kidney, Lv‐ liver, Sp‐ spleen, SM‐ skeletal muscle; Sig‐significant, Up‐ upregulated and Down‐ downregulated.

**Table S10:** Validation target genes and primers used for real‐time qPCR. A subset of DEGs in the bulk RNA‐seq datasets obtained from the liver, kidney, spleen and lung, were selected for validation by qPCR. Target genes were chosen based on their high expression levels and enrichment as hub genes among DEGs in any of the four organs. Abbreviations: Lv‐ liver, Kd‐ kidney, Sp‐ spleen, Lg‐ lung; Sig‐significant, Up‐ upregulated and Down‐ downregulated.

**Table S11:** Primers used for bulk RNA sequencing experiments. Reverse index primers used are 10 nt DNA indexes with 5 nt editing distance between any two indexes, as previously described by Hou et al. 2015 (Hou et al. 2015).

## Data Availability

The data that support the findings of this study are openly available in GEO Repository at https://www.ncbi.nlm.nih.gov/geo/query/acc.cgi?acc = GSE291420, reference number GSE291420.
