# Supplementary figures and images for: Deciphering the Transcriptomic Signatures of Aging Across Organs in Mice
Source: Aging Cell. 2026 Jan 8;25(2):e70357. doi: 10.1111/acel.70357 (PMC12783705; doi:10.1111/acel.70357)

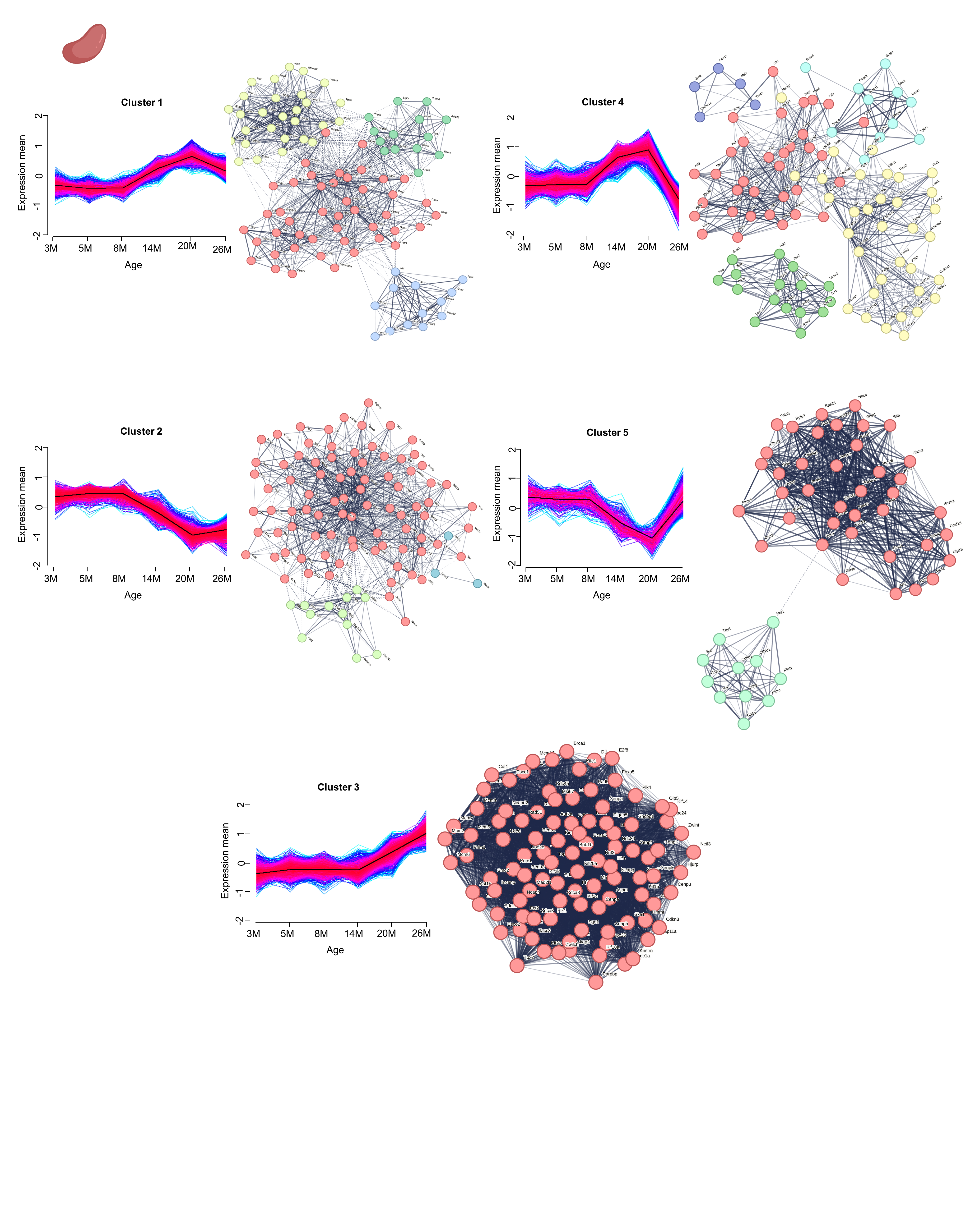

Supplement: Supplementary file 1 — Figure S1: Temporal gene expression clusters and hub gene networks in the spleen. Mfuzz clustering of z‐scored expression profiles of age‐associated differentially expressed genes (DEGs) in the spleen, illustrating distinct temporal trajectories. Each panel depicts the average cluster trend (bold line) alongside individual gene profiles. Adjacent to each trajectory plot, protein–protein interaction networks of the highest‐ranked hub genes are shown. Hub genes were defined by maximal clique centrality (MCC) scores using CytoHubba and visualized in STRING based on STRING interaction data. Nodes represent genes and edges denote functional associations, with densely connected nodes (“hubs”) indicating potential key regulators. Top 100 genes per cluster are shown except for cluster 5 where we show the top 50 gene hubs. [file ACEL-25-e70357-s016.tif]

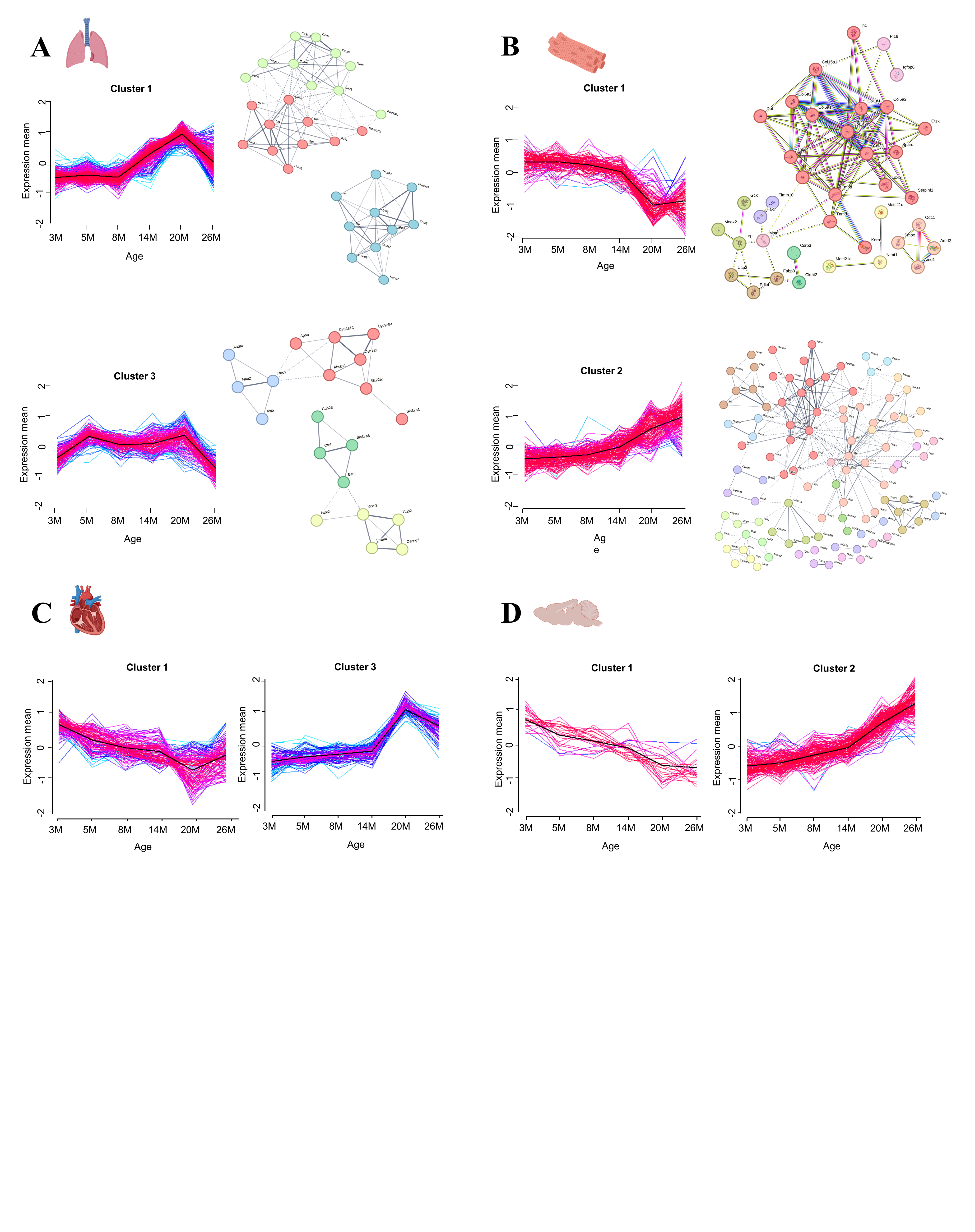

Supplement: Supplementary file 2 — Figure S2: Age‐associated gene expression trajectories and Organ‐Specific Transcriptomic Directionality (A–D) Mfuzz clustering of z‐scored expression profiles of age‐associated differentially expressed genes (DEGs) in lung (A), skeletal muscle (B), heart (C), and brain (D), revealing organ‐specific temporal trajectories during aging. Each cluster panel shows the mean expression trend of individual gene profiles. Protein–protein interaction networks of top hub genes are displayed adjacent to cluster plots, except for heart and brain, where the low number of DEGs precluded network construction. Hub genes were ranked by maximal clique centrality (MCC) using CytoHubba and visualized in STRING based on STRING interaction data. Nodes represent genes and edges indicate functional associations, with highly connected “hubs” potentially serving as key regulatory points. For network visualization, the following hub gene sets were used: lung ‐ top 30 genes for cluster 1 and top 20 for cluster 3; skeletal muscle ‐ all genes in clusters 1 and 2. (E) Tissue‐specific directionality of age‐related transcriptional changes derived from linear mixed‐effects modeling (LMM). Heatmap showing genes with significant Age‐by‐Tissue interaction effects (LMM, adjusted p < 0.05), followed by linear trend tests within each organ to determine directionality. Rows represent individual genes and columns represent organs. Colors indicate age‐associated expression changes: red = upregulated, blue = downregulated, and white = not significantly changed within each tissue. The matrix highlights both consistent and divergent aging trajectories across organs. [file ACEL-25-e70357-s010.tif]

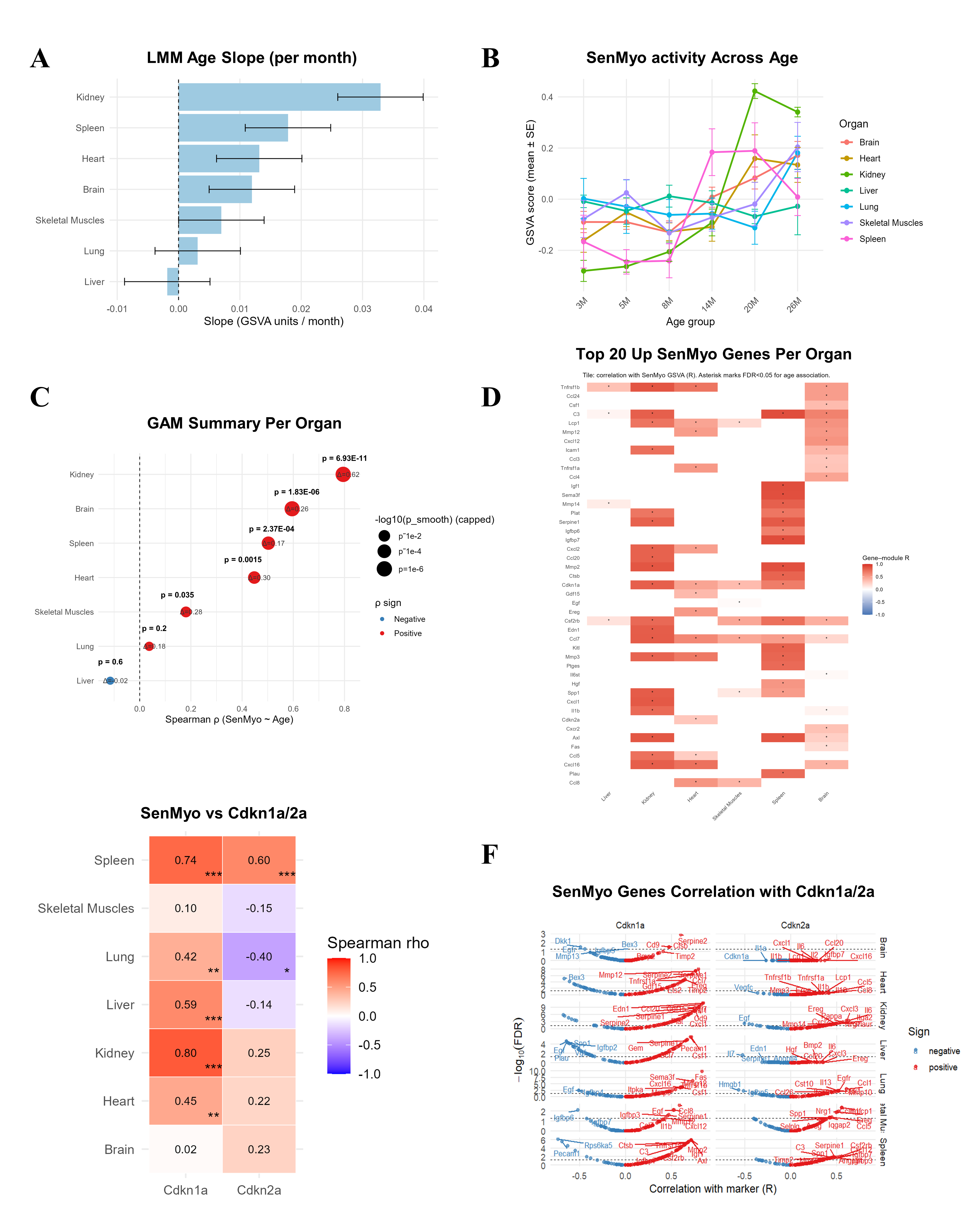

Supplement: Supplementary file 3 — Figure S3: Organ‐specific dynamics and drivers of the senescence/myofibroblast program (SenMyo) (A) LMM age slopes (per month). Estimated age effects of SenMyo activity (GSVA score) from a linear mixed model: SenMyo ~ Age × Organ + (1|MouseID). Bars show slope (GSVA units/month) ±95% CI from ‘emtrends’. Kidney and spleen show the steepest increases; liver is ~0/slightly negative. (B) SenMyo activity across age. Mean (±SE) SenMyo GSVA scores per age group and organ, computed with GSVA (Gaussian kernel) on the full transcriptome within each organ. Trajectories highlight early/strong rise in kidney, later/moderate rises in spleen/heart/brain, late shift in skeletal muscle, and flat profiles in lung and liver. (C) GAM summary per organ. For each organ, a GAM was fit to SenMyo vs. age (GAM: SenMyo ~ s(Age), REML). Point position = Spearman ρ (SenMyo vs. age); point size = −log10(p for the smooth term; capped for display); color encodes the sign of ρ; labels show Δ = mean change (26 M–3 M). (D) Heatmap of the top 20 age‐upregulated genes per organ that are coherent with the module. Each gene met (i) significant gene–age association (Spearman FDR < 0.05 within organ) and (ii) positive coherence with SenMyo (Pearson R between gene expression and SenMyo across samples); ranking = |R| × |ρ_age| × −log10(FDR_age). (E) Heatmap of Spearman correlations between SenMyo GSVA and Cdkn1a or Cdkn2a (p16) per organ. (F) Per‐gene Pearson correlations with markers. For each organ and marker (columns within facets), scatter of gene–marker Pearson correlations (R, x‐axis) vs. –log10(FDR) for the gene's age association (y‐axis). Points are colored by correlation sign (red = positive, blue = negative); select genes are labeled. [file ACEL-25-e70357-s014.tif]

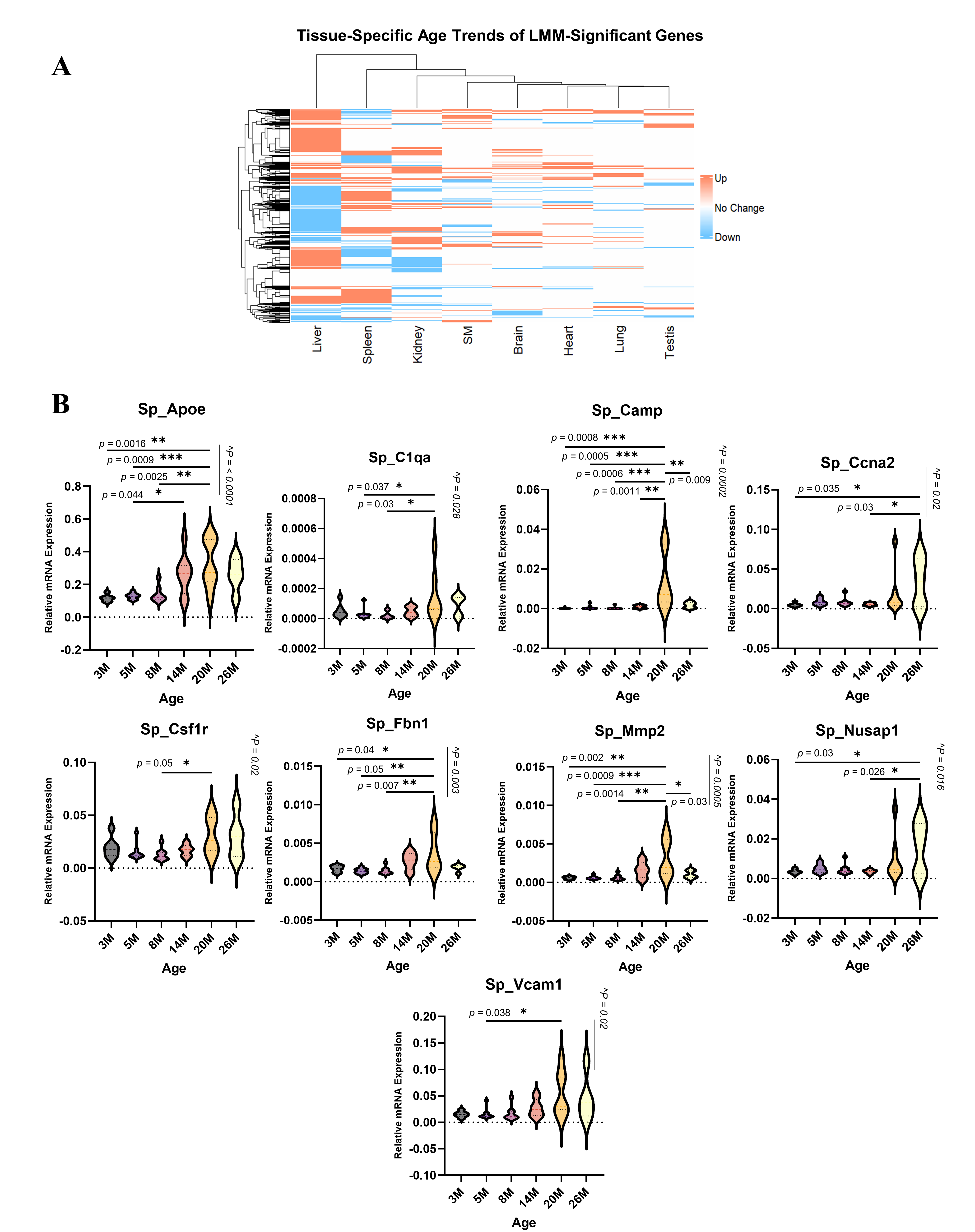

Supplement: Supplementary file 4 — Figure S4: Validation of age‐associated gene expression changes in the spleen. RT–qPCR analysis of selected aging‐associated genes in the spleen across different age groups. Gene expression levels were normalized to β‐actin using the 2−ΔCT method. Violin plots display the distribution of relative mRNA expression per group, with mean ± SEM indicated. Violin plot shows upper and lower quartiles (lightly dotted lines) and the median (bold dotted line). Statistical analysis was performed using one‐way ANOVA with age as a between‐subjects factor, followed by Tukey's post hoc test. Significance levels: *p < 0.05; **p < 0.01; ***p < 0.001; ****p < 0.0001. ^denotes ANOVA p value indicating overall age effect. [file ACEL-25-e70357-s005.tif]

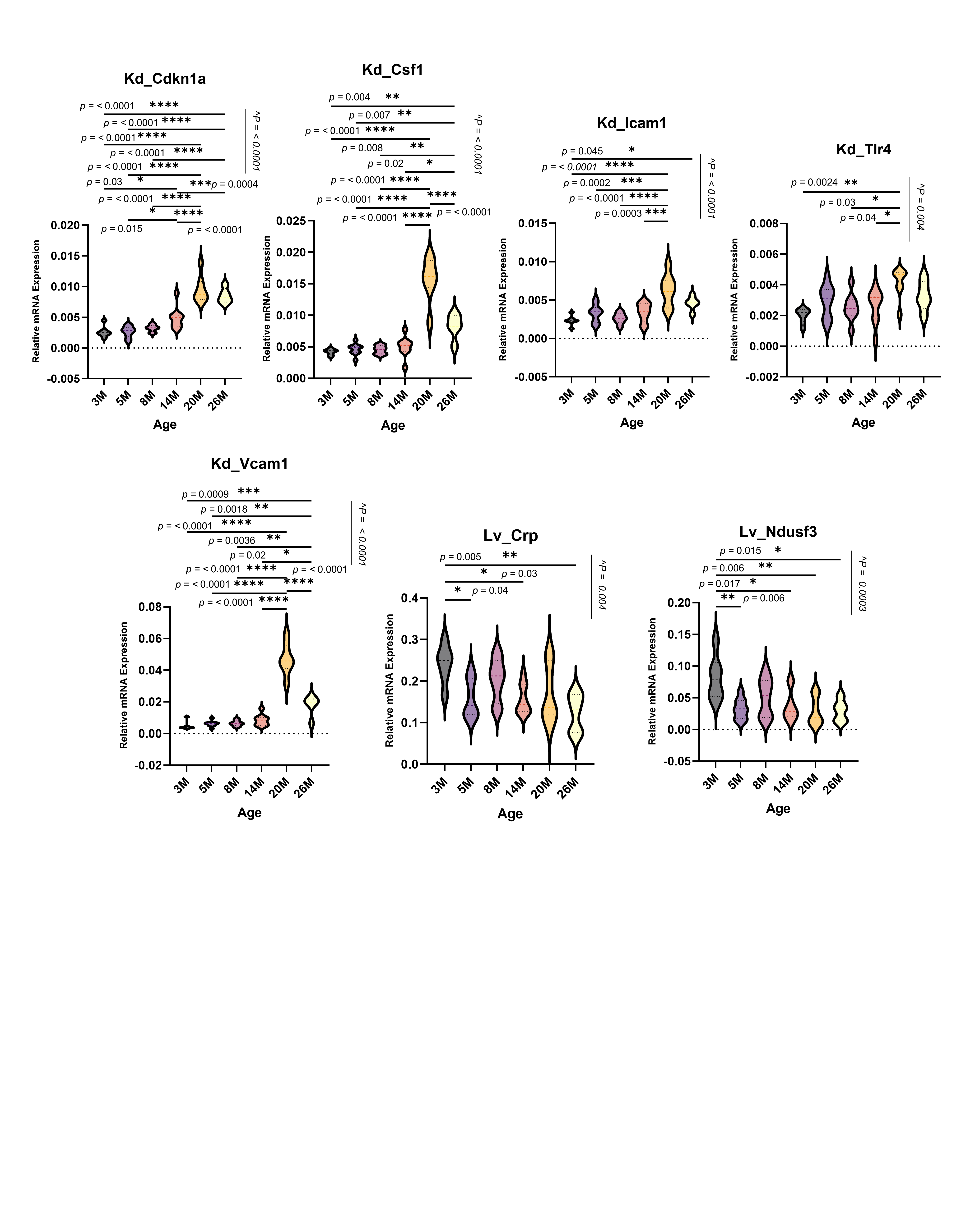

Supplement: Supplementary file 5 — Figure S5: Validation of age‐associated gene expression changes in the kidney and liver. RT–qPCR analysis of selected aging‐associated genes in the kidney and liver across different age groups. Gene expression levels were normalized to β‐actin using the 2−ΔCT method. Violin plots display the distribution of relative mRNA expression per group, with mean ± SEM indicated. Violin plot shows upper and lower quartiles (lightly dotted lines) and the median (bold dotted line). Statistical analysis was performed using one‐way ANOVA with age as a between‐subjects factor, followed by Tukey's post hoc test. Significance levels: *p < 0.05; **p < 0.01; ***p < 0.001; ****p < 0.0001. ^denotes ANOVA p value indicating overall age effect. [file ACEL-25-e70357-s012.tif]
